# Mitochondrial ecosystem restoration in Alzheimer’s disease: from mechanisms to multi-target therapeutic strategies

**DOI:** 10.3389/fcell.2026.1886145

**Published:** 2026-08-27

**Authors:** Dandan Song

**Affiliations:** Department of Pharmacy, Shaoxing Seventh People’s Hospital (Affiliated Mental Health Center, Shaoxing University), Shaoxing, China

**Keywords:** Alzheimer’s disease, mitochondrial dysfunction, mitochondrial ecosystem restoration, mitochondrial targeted therapy, TPP+ Conjugates

## Abstract

Alzheimer’s disease (AD), the most prevalent cause of dementia, lacks definitive cures despite decades of research focused on amyloid-beta (Aβ) and tau pathologies. Emerging evidence positions mitochondrial dysfunction not merely as a downstream consequence, but as the epicenter linking aging, metabolic failure, and neuroinflammation in AD pathogenesis. This study synthesizes the latest advances in mitochondrial-targeted therapies, framing them within a “Mitochondrial Ecological Restoration” perspective. I analyze the molecular mechanisms by which mitochondrial-targeted therapies modulate oxidative stress, mitochondrial dynamics, mitophagy and neuroinflammation, and evaluate their translational potential. Accumulating evidence indicates that strategies ranging from antioxidants (e.g., MitoQ) to mitophagy enhancement (e.g., Spautin-1) and biogenesis activation (e.g., PGC-1α Activator) have demonstrated efficacy in preclinical models. These interventions theoretically interrupt the pathological cycle between proteotoxicity and bioenergetic crisis. While challenges in blood-brain barrier (BBB) penetration and target specificity persist, the field is shifting from single-target scavenging to combinatorial ecosystem repair. Future success will require precise delivery systems, early biomarkers, and a paradigm shift toward treating the neuron as a metabolic ecosystem, though substantial translational challenges remain.

## 1 Introduction

Alzheimer’s disease (AD) is a complex neurodegenerative disorder characterized by a multifaceted pathological network. The Aβ cascade hypothesis is one of the most widely accepted pathological mechanisms. This hypothesis posits that the overproduction and deposition of beta-amyloid protein (Aβ) are the core pathological events in AD. Aβ is generated by the cleavage of amyloid precursor protein (APP) by β-secretase and γ-secretase, and its aggregation forms senile plaques, leading to neuronal toxicity (Abduljawad et al., 2025; Sciaccaluga et al., 2021). Elevated Aβ42/Aβ40 ratio favors Aβ aggregation into neurotoxic amyloid fibrils and the induction of tau protein pathology, ultimately causing neuronal cell death and neurodegeneration (Breijyeh and Karaman, 2020).

AD is now increasingly understood as a complex, multifactorial pathological network rather than a linear cascade driven by a single initiating event. The core pathological hallmarks include extracellular amyloid-beta plaques, intracellular neurofibrillary tangles composed of hyperphosphorylated tau, chronic neuroinflammation, and progressive synaptic and neuronal loss. These hallmarks are interconnected through bidirectional feedforward loops that amplify each other over decades of prodromal progression. Aging remains the single greatest risk factor, lowering the threshold for protein aggregation, impairing cellular clearance mechanisms, and sensitizing the brain to metabolic and inflammatory insults. Within this network, mitochondrial dysfunction has emerged as a critical node that both responds to and exacerbates amyloid-beta and tau pathologies, while also serving as a major source of neuroinflammatory signals via damage-associated molecular patterns. However, the relative contribution of each pathological driver likely varies across disease stages, brain regions, and individual genetic backgrounds, a heterogeneity that complicates the search for universal therapeutic targets and underscores the need for multi-pronged intervention strategies.

Recent Phase III clinical trials have refined this view. Both lecanemab (Clarity AD) and donanemab (TRAILBLAZER-ALZ 2) have demonstrated that monoclonal antibodies targeting aggregated Aβ can modestly but statistically significantly slow cognitive decline in patients with early-stage AD ((van Dyck et al., 2023)) (Sims et al., 2023). This suggests that Aβ is not merely a bystander but one of the key drivers in the disease process. Nevertheless, the limited magnitude and transient nature of these benefits underscore that Aβ clearance alone is insufficient to halt the broader neurodegenerative cascade, implying the existence of upstream or parallel pathogenic drivers.

Against this backdrop, the mitochondrial cascade hypothesis has emerged as a vital complementary theoretical framework to the amyloid cascade hypothesis. Classical mitochondrial cascade hypothesis was first proposed, which posits that genetic and aging-induced mitochondrial dysfunction serves as an upstream initiating factor in sporadic Alzheimer’s disease (SAD), occurring prior to Aβ deposition and tau protein hyperphosphorylation (Swerdlow and Khan, 2004). Subsequently, Manczak and Reddy et al. further demonstrated that Aβ can specifically aggregate within mitochondria, directly impairing electron transport chain function and amplifying oxidative stress and neuroinflammation (Manczak et al., 2006), as well as elaborating on how mitochondrial dynamics and mitochondrial biogenesis abnormalities in AD mediate synaptic damage and cognitive decline (Manczak et al., 2011). Collectively, these studies establish the mitochondrion as a hub in AD pathology. It must be acknowledged, however, that the mitochondrial cascade hypothesis does not fully account for all aspects of Alzheimer’s disease pathogenesis. The temporal sequence between mitochondrial dysfunction and the appearance of amyloid-beta plaques or tau tangles remains a subject of debate, with evidence supporting both mitochondria-driven pathology and pathology-driven mitochondrial damage depending on the experimental model and disease stage. Moreover, the heterogeneity of sporadic Alzheimer’s disease, influenced by aging, vascular factors, systemic metabolism, and genetic variants beyond APOE, suggests that mitochondrial dysfunction may play a more prominent upstream role in some patient subgroups than in others. Therefore, rather than positioning mitochondria as the exclusive upstream trigger, a more nuanced view recognizes that mitochondrial dysfunction, amyloid-beta, tau, and neuroinflammation operate within a self-reinforcing network in which each node can amplify the others.

The “mitochondrial cascade hypothesis” positions organelle dysfunction as the upstream initiator of AD, preceding Aβ deposition and neurofibrillary tangle formation. Rather than merely serving as “energy factories,” mitochondria act as the central hubs integrating metabolic signaling, redox balance, and innate immune responses. In AD, a vicious cycle is established where bioenergetic failure amplifies oxidative stress, which in turn damages mitochondrial DNA (mtDNA), triggering a neuroinflammatory storm via pattern recognition receptors. The fine structure of mitochondria, particularly the mitochondrial contact sites and cristae organizing system (MICOS) responsible for forming inner membrane cristae, undergoes significant alterations in AD (9). Recent studies have found that mitochondrial dysfunction is closely linked to these hypotheses mentioned above. In the context of aging factors and AD risk, increased mitochondrial Aβ42 is associated with exacerbated synaptic mitochondrial dysfunction in mouse model expressing nonmutant humanized Aβ (humanized Aβ-knockin [hAβ-KI] mice) (Jia et al., 2023a). In AD animal models, the expression of human total tau or phosphorylated tau leads to mitochondrial dysfunction (Liu et al., 2023). Meanwhile, the accumulated pathological proteins will further damage the mitochondria, forming a self-amplifying vicious cycle, which ultimately leads to widespread neuronal degeneration.

Although a substantial number of studies have investigated the relationship between mitochondrial dysfunction and AD, several unresolved issues remain further attention. Firstly, existing research is predominantly focused on the phenotypic description of mitochondrial dysfunction, with insufficient in-depth analysis of its specific molecular mechanisms. For instance, the specific signaling pathways through which mitochondrial dynamics abnormalities influence the pathological process of AD remain incompletely elucidated. Secondly, concerning the causal relationship between mitochondrial dysfunction and other pathological features of AD (such as Aβ deposition and tau protein hyperphosphorylation), current research findings are inconsistent and require further verification. Furthermore, while certain mitochondrial-targeted drugs have shown potential efficacy in cellular and animal models, their clinical translation remains hindered by numerous challenges, including issues related to drug delivery efficiency and target specificity.

Based on this, this review proposes a conceptual shift from single-target clearance to mitochondrial ecological restoration (MER). To operationalize this concept, I define MER as a multidimensional, phased, multi-target combined intervention therapeutic paradigm, with the core objective of systematically reconstructing the entire mitochondrial ecological homeostasis, rather than merely correcting a single isolated mitochondrial damage. To make this concept actionable for translational research, I propose a quantitative heuristic framework rather than a definitive clinical endpoint. I define MER as a multidimensional, phased, multi-target combined intervention paradigm aimed at systematically reconstructing the entire mitochondrial ecological homeostasis. Operationally, we consider MER to be achieved when at least three of the following five quantitative criteria are simultaneously met relative to pre-treatment baseline levels: 1. Mitochondrial Bioenergetics; 2. Mitochondrial Redox; 3. Mitochondrial Dynamics; 4. Mitochondrial Quality Control; 5. Metabolism-Immune Axis. I acknowledge that the specific normalization thresholds for each biomarker, for example, a 30% reduction in 8-OHdG, currently lack experimental calibration. Therefore, I propose this ≥3/5 rule as a testable operational definition intended to guide preclinical combination studies. This ≥3/5 rule is a provisional heuristic hypothesis rather than validated clinical cut-off values, proposed only to guide preclinical combinatorial drug screening. No large-scale human cohort or multi-animal model data has calibrated the exact normalization threshold of each biomarker. I select the threshold of at least three recovered indicators for two rationales: First, single or dual index improvement only partially relieves local mitochondrial injury and cannot break the bidirectional pathological vicious cycle among bioenergetics, redox and inflammation; Second, simultaneous normalization of all five dimensions is difficult to achieve with current single or dual drug regimens in preclinical models, making ≥3 a pragmatic intermediate standard for preliminary therapeutic evaluation. This numerical cutoff remains flexible and open to future experimental adjustment. Future research should employ receiver operating characteristic analysis to determine the optimal biomarker cutoffs that best correlate with cognitive endpoints in patient populations. This framework distinguishes MER from traditional “mitochondrial rescue” by emphasizing the systemic rewiring of the mitochondrial ecosystem rather than isolated pathway correction. Specifically, this review aims to: Systematically synthesize molecular mechanisms underlying mitochondrial dysfunction in AD; Critically evaluate the translational potential of current mitochondrial-targeted therapeutic strategies; Propose an actionable MER framework to guide future combinatorial and precision intervention paradigms. Clearly distinguishing MER from related concepts is essential to avoid terminological ambiguity. MER differs from the classical mitochondrial cascade hypothesis, which positions mitochondrial dysfunction as a unidirectional upstream initiator of Aβ pathology. Instead, MER regards mitochondria as a dynamic ecosystem where the pathological network operates through bidirectional, self-amplifying loops between bioenergetic failure, proteotoxicity, and innate immune activation. Whereas mitochondrial quality control, or MQC, primarily targets the selective degradation of damaged individual organelles through fission, fusion, and mitophagy, MER encompasses a broader framework that includes inter-organelle network rewiring and the metabolic-immune interface. In contrast to the mitochondrial cascade hypothesis, which assumes a linear and unidirectional pathogenic progression, MER adopts a systems-network perspective that emphasizes reciprocal interactions between mitochondrial populations and their surrounding cellular microenvironment. Moreover, MER is fundamentally distinct from mitohormesis, which relies on adaptive stress responses to low-dose insults; instead, MER pursues active and targeted reconstruction of endogenous mitochondrial networks. Finally, unlike mitochondrial transplantation, a cell-replacement strategy, MER focuses on revitalizing the host’s native mitochondrial pool. Operationally, this phased intervention logic, comprising clearance, reconstruction, and consolidation, distinguishes MER from conventional *ad hoc* multi-target strategies and provides a temporally coordinated roadmap for therapeutic design.

## 2 Mitochondrial dysfunction in Alzheimer's disease

### 2.1 The structural collapse: from MICOS to dynamics

**FIGURE 1:**
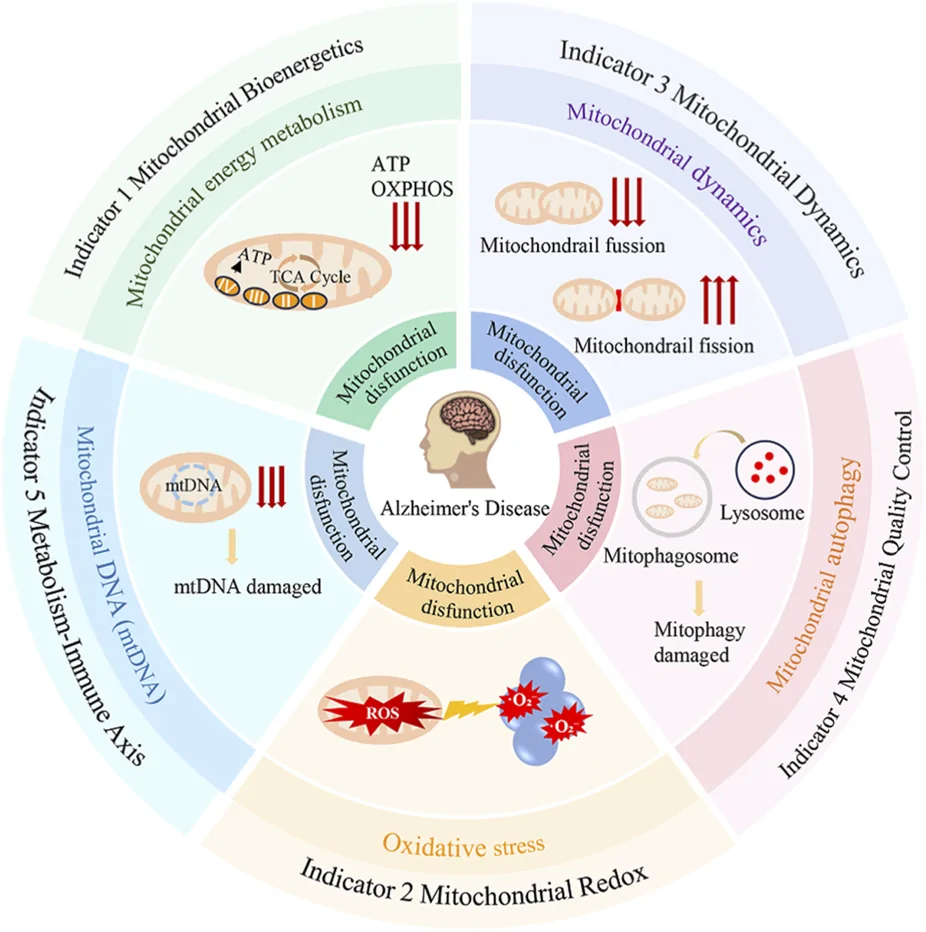
Mitochondrial dysfunction in AD. All pathway labels and pathological indicators in this figure represent well-documented mitochondrial lesions validated by multiple independent preclinical and human brain tissue studies, which constitute established evidence of AD pathogenesis. The specific manifestations of mitochondrial dysfunction leading to AD mainly include excessive production of reactive oxygen species (ROS), impaired generation of new mitochondria and excessive fission, mitochondrial autophagy impairment, damage and abnormalities of mitochondrial DNA (mtDNA) and reduction of ATP synthesis. These five pathological dimensions exactly correspond to the five quantitative evaluation criteria proposed of MER. Unlike conventional single-target mitochondrial interventions that only correct one isolated lesion, the MER paradigm advocates synchronous systematic restoration of the interconnected mitochondrial compartments shown in this figure to break the self-amplifying pathological vicious cycle underlying AD neurodegeneration.

The physical architecture of mitochondria is the foundation of their function. In AD, the structural integrity of the mitochondrial inner membrane is compromised at the molecular level, leading to a bioenergetic crisis (Figure 1).

#### 2.1.1 Cristae disorganization and MICOS destabilization

As the ‘energy factories’ of cells, mitochondria exhibit a significant decline in energy production capacity in AD, an imbalance in bioenergetic metabolism that represents an important feature of early disease. The electrical physiological activity of brain neurons mainly depends on ATP provided by mitochondrial oxidative phosphorylation (Li et al., 2022).

In AD patient brains, MIC60 (IMMT) expression is halved relative to controls (Sathe et al., 2021). Defective MICOS disrupts mitochondrial cristae and oxidative phosphorylation (OXPHOS) efficiency (Wang et al., 2025a), accompanied by suppressed Complex III/IV activity in the hippocampus and temporal cortex (Li et al., 2022). This impairs ATP synthesis and triggers neuronal energy deficiency; ATP concentration and the NAD^+^/NADH ratio act as core biomarkers for MER efficacy. Meanwhile, AD disrupts glucose metabolic networks: reduced pyruvate dehydrogenase (PDH) and two-oxoglutarate dehydrogenase (2-OGDH) activity lowers TCA intermediate pools (e.g., glutamate, GABA), impairing neurotransmitter production and neuronal function (Li et al., 2022).

#### 2.1.2 Dynamic abnormalities: excessive fission and fusion defects

Mitochondrial dynamics, regulated by fission (Drp1) and fusion (Mfn1/2, OPA1) proteins, maintain a healthy network. In AD, this balance is skewed towards excessive fission. Pathological levels of Drp1 lead to mitochondrial fragmentation, a hallmark of early AD. This fragmentation not only disrupts the mitochondrial network but also facilitates the trafficking of toxic Aβ oligomers to synapses (Baek et al., 2017). In AD, the expression of mitochondrial fusion proteins Mfn2 and OPA1 is significantly reduced in many studies. This reduction, together with increased expression or activity of fission proteins such as Drp1, is widely considered a hallmark of mitochondrial dynamics imbalance, though the relative contribution of fusion versus fission defects may vary across neuronal subtypes and disease stages. (Keskinoz et al., 2025). The direct consequence of this imbalance is excessive fragmentation of the mitochondrial network, and the reduction in fusion proteins means that cells lose critical internal repair and defense mechanisms.

### 2.2 Quality control failure: the vicious cycle of mitophagy and biogenesis

Beyond structural defects, AD is characterized by a failure in mitochondrial quality control (MQC), where the clearance of damaged organelles fails to keep pace with the generation of new ones.

#### 2.2.1 Mitophagy blockade: the accumulation of damaged units

Mitophagy is the process by which lysosomes selectively degrade dysfunctional mitochondria, which is crucial for maintaining mitochondrial health, regulating mitochondrial quantity, and preventing reactive oxygen species (ROS) and cellular stress (Wang et al., 2023; Kan Yang et al., 2024). Under AD pathological conditions, mitophagy is significantly inhibited. This diminished clearance capacity results in the accumulation of dysfunctional mitochondria within cells, which release large amounts of harmful substances and exacerbate neuronal damage (QIN et al., 2025). Serine/threonine PTEN-induced putative kinase 1(PINK1) functions as a mitochondrial damage sensor, Parkin acts as a signal amplifier, and ubiquitin chains serve as signal effectors. Collectively, these components determine how damaged mitochondria initiate mitophagy (Harper et al., 2018). In AD, the PINK1/Parkin pathway-mediated mitochondrial autophagy is inhibited, leading to accumulation of damaged mitochondria and triggering oxidative stress and inflammatory responses (McGill Percy et al., 2025).

Abnormalities in mitophagy (such as levels of PINK1/Parkin pathway proteins) and dynamics (such as the phosphorylation/cleavage status of Drp1 and OPA1) are early events in AD. By detecting the expression or activity of these key regulatory proteins, the capacity for mitochondrial self-renewal and remodeling can be assessed. The restoration of stable PINK1 expression or the correction of excessive Drp1 activation should serve as molecular evidence for the efficacy of corresponding targeted therapies.

#### 2.2.2 Biogenesis failure: the diminished regenerative capacity

Mitochondrial biogenesis is a coordinated transcriptional program responsible for synthesizing intact functional mitochondria. This process is orchestrated by peroxisome proliferator-activated receptor gamma coactivator 1-alpha (PGC-1α), which serves as the master coordinator. PGC-1α activates downstream transcription factors including nuclear respiratory factor 1 and 2 (NRF1 and NRF2), which in turn upregulate the expression of mitochondrial transcription factor A (TFAM). TFAM is essential for the replication, transcription, and maintenance of mitochondrial DNA (mtDNA) (Clemente-Suárez et al., 2023). In AD, PGC-1α expression is significantly reduced, leading to decreased mtDNA copy number, reduced transcription of mtDNA-encoded electron transport chain subunits, and diminished mitochondrial mass. This failure to replenish the mitochondrial pool results in a progressive decline in ATP synthesis capacity and an inability to compensate for ongoing mitochondrial damage (Clemente-Suárez et al., 2023).

### 2.3 The metabolic-immune axis: mtDNA as DAMPs

**FIGURE 2:**
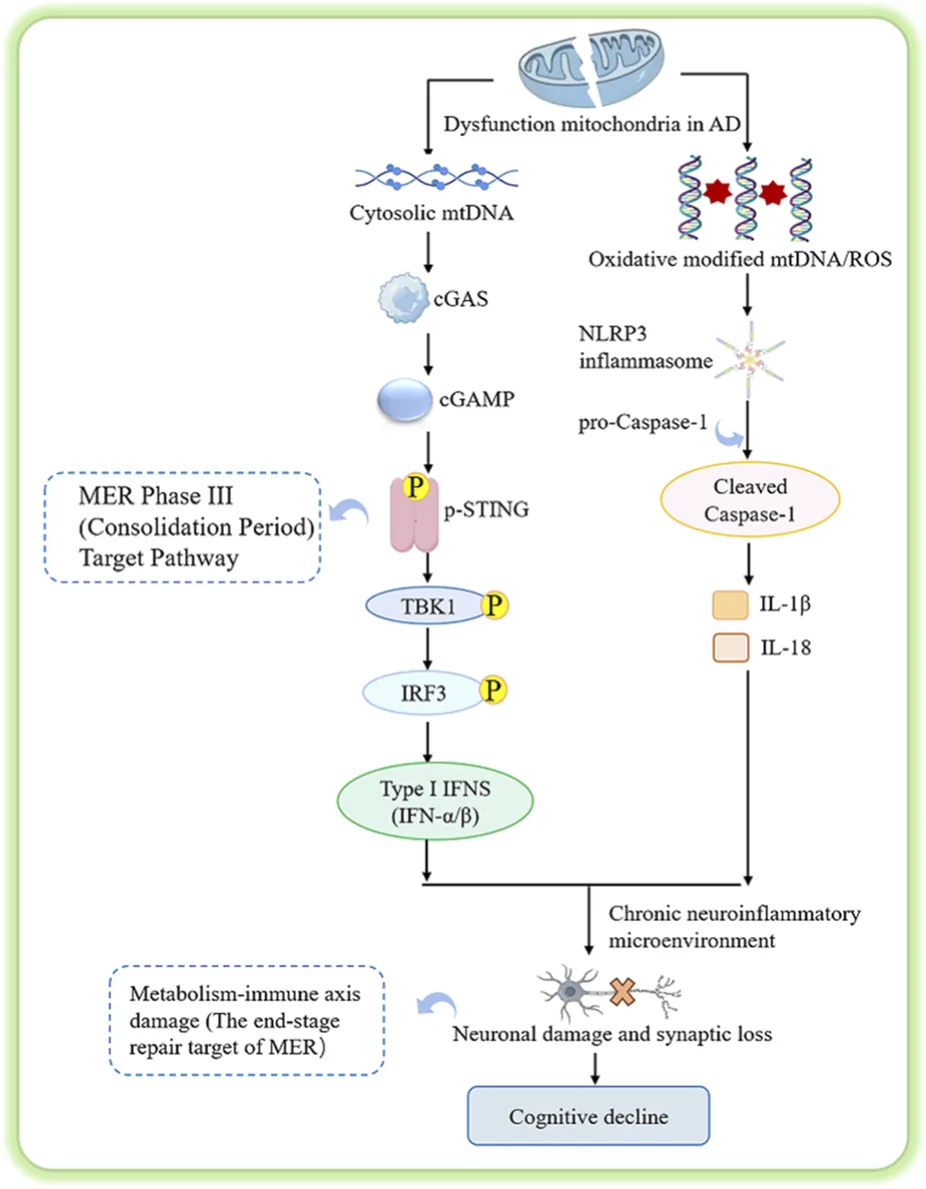
Mitochondrial dysfunction drives innate immune activation and neuroinflammation in AD. The black solid-line arrows denote established pathological signaling pathways that have been validated by multiple independent studies, including mitochondrial DNA release, the cGAS-STING pathway, and NLRP3 inflammasome activation. The Phase III of MER (consolidation period), enclosed within the blue dashed box, represents a provisional therapeutic hypothesis proposed in this review and has not yet been systematically validated experimentally. In AD, damaged mtDNA into the cytosol is recognized by cyclic GMP-AMP synthase (cGAS), which produces cGAMP to activate the stimulator of interferon genes (STING). Activated STING recruits and phosphorylates TBK1, which in turn phosphorylates the transcription factor IRF3, leading to the expression of type I interferons (IFN-α/β). Oxidative modified mtDNA and ROS directly activate the NLRP3 inflammasome, which promotes the cleavage of pro-caspase-1 into active caspase-1 and subsequently cleaves pro-IL-1β and pro-IL-18 into their mature forms. The convergence of these pathways establishes a chronic neuroinflammatory microenvironment, ultimately driving neuronal damage, synaptic loss, and cognitive decline. mtDNA release-mediated innate immune pathways constitute the pathological circuit of the metabolic-immune axis within the MER framework. This pathway is a key intervention target during the phase III (Consolidation Period) of MER triple therapy.

One of the most compelling recent advances is the recognition of mitochondria as immune regulators. In AD, damaged mitochondria release mtDNA into the cytosol, transforming a metabolic organelle into an immune activator (Brooks et al., 2026) (Figure 2).

#### 2.3.1 Oxidative stress and excessive production of reactive oxygen species (ROS)

Impaired electron transport chain complexes I and III cause electron leakage, leading to excessive mitochondrial ROS production, including superoxide anions. These ROS attack lipids, proteins, and DNA, causing widespread oxidative damage and further impairing mitochondrial function, establishing a self-amplifying destructive cycle (McGill Percy et al., 2025). Concurrently, ROS impair calcium transport proteins, triggering mitochondrial calcium overload, which exacerbates membrane potential loss and permeability transition pore opening, ultimately resulting in mitochondrial outer membrane rupture (Wang et al., 2024; Zhang et al., 2023). This vicious cycle of oxidative damage and calcium overload represents a core mechanism of neuronal injury in AD.

Direct detection of ROS presents technical challenges, however, the oxidative damage they induce serves as a stable biomarker. Lipid peroxidation products (such as 4-HNE and MDA) and DNA oxidation products (such as 8-OHdG) are often elevated in the cerebrospinal fluid and peripheral blood of AD patients. They not only serve as indirect evidence of mitochondrial oxidative stress, but changes in their levels can also reflect whether antioxidant therapies effectively eliminate free radicals originating from mitochondria.

#### 2.3.2 mtDNA release and innate immune activation

Mitochondria possess independent genetic material mtDNA, which exhibits significant abnormalities in AD. Cytosolic mtDNA is recognized by cyclic GMP-AMP synthase (cGAS), which catalyzes cGAMP production to activate the STING-TBK1-IRF3 signaling axis, driving type I interferon expression (Li et al., 2021a; Zhang et al., 2026). Oxidative modified mtDNA and ROS directly activate the NLRP3 inflammasome, which promotes the cleavage of pro-caspase-1 into active caspase-1 and subsequently cleaves pro-IL-1β and pro-IL-18 into their mature forms (Shimada et al., 2012) (Jauhari et al., 2026). Both pathways are chronically activated in AD and collectively constitute key drivers of sustained neuroinflammation. Extracellularly released mtDNA activates Toll-like receptor 9 (TLR9) on immune cells, promoting neutrophil migration and inflammatory mediator release. The convergence of these pathways establishes a chronic neuroinflammatory microenvironment, ultimately driving neuronal damage, synaptic loss, and cognitive decline.

MtDNA resides adjacent to ROS generation sites with limited repair machinery, rendering it prone to oxidative lesions and mutations; reduced mtDNA copy number (mtDNA-CN) causally promotes AD pathogenesis (Wang et al., 2024). Mitochondrial haplogroups, shaped by stable mtDNA variants, drive interindividual disparities in mtDNA stability, basal ROS output and DAMP release, serving critical AD risk modifiers independent of APOE ε4 ((Marom et al., 2017)). Cross-population meta-analyses show carriers of H/HV/J haplogroups face lower AD risk and milder cognitive deterioration, while T/U/K haplotypes confer elevated vulnerability. The Utah prospective cohort further validates robust neuroprotection of H6A1A/H6A1B subhaplogroups against progression from mild cognitive impairment to AD ((Ridge et al., 2012)). Notably, the J haplogroup displays subtype heterogeneity reported in ADNI cohorts: European mainstream J variants exert protective effects, yet subclones with OXPHOS mutations aggravate hippocampal atrophy, hypometabolism and rapid cognitive decline (Liu et al., 2021a). Transmitochondrial cybrid assays mechanistically explain this duality: J cells generate low basal ROS but undergo severe electron leakage, massive ROS burst, mtDNA fragmentation and hyperinflammation upon Aβ or calcium stress, whereas H haplotypes sustain intact respiratory chains to limit oxidative injury and mtDNA efflux (Panvini et al., 2022; Atilano et al., 2015).

ROS and mtDNA, as a damage-associated molecular patterns (DAMPs), collectively constitute a core pathological axis. ROS is the culprit responsible for initiating and exacerbating mtDNA damage and its release, and the release of mtDNA converts intracellular metabolic crises and oxidative damage into potent immune signals that drive local and systemic inflammation. In neurodegenerative diseases, the sustained activation of this axis is considered a key mechanism underlying chronic neuroinflammation and progressive neuronal loss. Future intervention strategies may not be limited to traditional antioxidant (ROS scavenging) or anti-inflammatory approaches, but rather require a dual-pronged approach or disruption of this cycle.

In various diseases such as AD, a reduction in peripheral blood mtDNA copy number (mtDNA-CN) is often associated with decreased mitochondrial content and functional decline. Consequently, mtDNA-CN may serve as a potential biomarker for evaluating the efficacy of mitochondrial biogenesis-activating therapies, such as SIRT1/PGC-1α pathway modulators.

### 2.4 The inter-organellar axis: mitochondria-associated ER membranes (MAMs), calcium homeostasis, and mitochondrial proteostasis

Mitochondria do not function in isolation; they are physically and functionally tethered to the endoplasmic reticulum, or ER, through specialized membrane contact sites known as mitochondria-associated ER membranes, or MAMs. These dynamic platforms serve as critical hubs for lipid synthesis, calcium transfer, and autophagosome formation. Their disruption in AD has emerged as an early pathogenic event that precedes overt Aβ deposition.

#### 2.4.1 MAM dysfunction in AD

MAMs are structurally defined by tethering complexes that bring the ER and outer mitochondrial membrane into close apposition at a distance of 10–25 nm. Key MAM-resident proteins, including the inositol 1,4,5-trisphosphate receptors, or IP3Rs, the glucose-regulated protein 75, or GRP75, and the voltage-dependent anion channel 1, or VDAC1, form the core machinery for calcium transfer from ER stores to mitochondria. In Alzheimer’s disease, this tethering apparatus is profoundly altered. Elevated expression of TREM2, a microglial receptor enriched at MAMs, has been linked to altered lipid metabolism and enhanced Aβ production in early disease stages. Conversely, reduced expression of PACS2, a MAM-localized sorting protein, impairs ER-mitochondria communication and sensitizes neurons to apoptotic stimuli. Importantly, presenilin-1 and presenilin-2, the catalytic subunits of γ-secretase, are also enriched at MAMs, where they regulate IP3R-mediated calcium release. Familial Alzheimer’s disease-linked mutations in these presenilins disrupt this regulation, leading to exaggerated calcium transfer and mitochondrial calcium overload. This aberrant calcium flux is believed to promote mitochondrial permeability transition pore opening and cytochrome c release, thereby amplifying the apoptotic cascade in vulnerable neurons.

#### 2.4.2 Mitochondrial calcium dyshomeostasis

Mitochondrial calcium homeostasis is tightly controlled by the mitochondrial calcium uniporter complex, which mediates rapid calcium uptake across the inner membrane, and by the sodium-calcium exchanger, which facilitates calcium efflux. In Alzheimer’s disease, this balance is profoundly disturbed. Enhanced ER-to-mitochondria calcium transfer via hyperactive IP3R-GRP75-VDAC1 complexes, combined with impaired calcium efflux, results in mitochondrial calcium overload. Elevated matrix calcium directly stimulates dehydrogenases of the tricarboxylic acid cycle, transiently boosting ATP production; however, sustained overload triggers permeability transition pore opening, dissipates the mitochondrial membrane potential, and unleashes a cascade of reactive oxygen species generation and cytochrome c-mediated apoptosis. Moreover, calcium-dependent activation of calcineurin has been shown to dephosphorylate and activate Drp1, further promoting the excessive mitochondrial fission that characterizes neurons in AD. Preclinical studies suggest that pharmacological inhibition of the mitochondrial calcium uniporter or genetic ablation of its regulatory subunit MICU1 may attenuate calcium-induced mitochondrial damage, though these approaches remain experimental.

#### 2.4.3 Mitochondrial proteostasis and the unfolded protein response

Mitochondria harbor their own proteostatic machinery, including chaperones such as mtHSP70, HSP60, and HSP10, as well as proteases like LONP1 and CLPP, which maintain the folding and clearance of mitochondrial proteins. When proteotoxic stress exceeds the capacity of these quality control systems, the mitochondrial unfolded protein response, or UPRmt, is activated. This retrograde signaling pathway upregulates nuclear-encoded mitochondrial chaperones and proteases to restore proteostasis. In Alzheimer’s disease, UPRmt is impaired. The accumulation of misfolded proteins within mitochondria, exacerbated by oxidative damage to mitochondrial DNA and respiratory chain subunits, overwhelms the chaperone network. Reduced expression of LONP1 and CLPP has been observed in Alzheimer’s disease brains, correlating with increased levels of carbonylated proteins and respiratory chain dysfunction. Furthermore, the accumulation of amyloid-beta within mitochondria directly interferes with protein import machinery, including the translocase of the outer membrane complex, creating a vicious cycle in which Aβ impairs proteostasis and proteostasis failure accelerates Aβ toxicity. Recent evidence suggests that pharmacological activation of UPRmt, via agents such as oligomycin or specific small-molecule inducers, may restore mitochondrial proteostasis and improve bioenergetic function in Alzheimer’s disease models, although this strategy remains at an early stage of investigation.

#### 2.4.4 Integration with the neuroimmune axis and the MER framework

The inter-organellar disruptions described above are not isolated phenomena but converge on the same mtDNA-driven innate immune axis outlined in Section 2.3.

MAM dysfunction and calcium overload both sensitize mitochondria to permeability transition, which in turn promotes mtDNA release into the cytosol. Once released, mtDNA activates the cGAS-STING and NLRP3 inflammasome pathways, driving the neuroinflammatory response that sustains Alzheimer’s disease progression. Moreover, impaired mitochondrial proteostasis increases the availability of oxidized mtDNA, a particularly potent NLRP3 agonist, further amplifying inflammation. Thus, the MAM-calcium-proteostasis network functions as an upstream modulator of the metabolic-immune axis. An ecosystem-level restoration strategy must therefore address not only the mitochondria themselves but also their inter-organellar context. This expanded perspective reinforces the MER framework by highlighting that mitochondrial health cannot be uncoupled from the broader cellular microenvironment, and that successful therapeutic strategies will likely need to target multiple nodes across this interconnected network.

## 3 Mitochondrial targeted therapeutic strategies: from antioxidants to ecosystem engineering

Current mitochondrial targeted therapeutic can be categorized based on their restoration strategy. However, the future lies in moving beyond monotherapy (Table 1).

**TABLE 1:** Mitochondrial targeted therapeutic strategies and representative drug.

Target/Mechanism	Drugs or compounds	Principle of action	Toxicity, narrow therapeutic index, and off-target risk	Levels of evidence (tier I–IV)	Evidence priority	Blood-brain barrier penetrability	Current clinical development status
Antioxidant stress	MitoQ	Deliver antioxidants specifically to mitochondria to neutralize reactive oxygen species and mitigate oxidative proteotoxicity and lipoperoxidation	Narrow therapeutic index, high-dose pro-oxidative damage, mitochondrial non-specific binding	Tier I	Strong	Moderate	Completion of Phase II clinical trials for Parkinson’s Disease; AD clinical trials have not yet started
	SkQ1			Tier I	Strong	Moderate	Preclinical research
	SS-31 (Elamipretide)	Stabilize mitochondrial membranes, protect cardiolipin, reduce ROS production, and improve mitochondrial bioenergetics	Accumulation in adipose tissue or the liver leads to off-target binding of cardiolipin in peripheral normal mitochondria, disrupting cellular homeostasis	Tier I	Strong	Moderate to high	Preclinical research
Mitophagy	Spautin-1	Enhance mitophagy, clear of dysfunctional mitochondria selectively	Spautin-1: Off-targetly interferes with the ubiquitin pathway; a narrow therapeutic window UA: Prone to cause off-target excessive autophagy in intestinal mitochondria	Tier II	Moderate	Low	Preclinical research
	Urolithin A (UA)			Tier I	Strong	Moderate	Preclinical research
Mitochondrial biogenesis	Rhein	Activate SIRT1/PGC-1α pathway to promote the generation of new healthy mitochondria, replacing damaged mitochondria	Rhein: At high concentrations, it will off-target to inhibit mitochondria in the kidneys and liver NR: Overactivation of peripheral silent proteins; long-term safety boundaries unclear Resveratrol: Off-target effects on multiple kinases and histone modification pathways throughout the body; High-dose gastrointestinal toxicity	Tier II	Moderate	Low	Preclinical research
​	Nicotinamide Riboside (NR)			Tier I	Strong	Moderate	Preclinical research
	Resveratrol			Tier III	Disappointing Clinical	Low	Completion of Phase II clinical trials for mild to moderate AD
Mitochondrial dynamics	Mdivi-1	Inhibit excessive mitochondrial fission, promote mitochondrial fusion, and maintain network integrity	Inhibiting systemic physiological mitochondrial fission; off-target disruption of basal quality control; extremely narrow therapeutic window	Tier II	Moderate	Low	Preclinical research
	P110			Tier II	Moderate	Low	Preclinical research
​	Coenzyme Q10 (CoQ10)	Improve the structural and functional integrity of mitochondria, regulate mitochondrial membrane potential, and promote mitochondrial biogenesis, thereby enhancing mitochondrial oxidative phosphorylation efficiency	Imbalance of peripheral redox homeostasis; narrow effective dose range	Tier III	Disappointing Clinical	Low	Termination of Phase III clinical trials
​	Curcumin	Alleviate the progression of AD through the JMJD3-H3K27me3-BDNF axis, helping maintain the balance of the mitochondrial stress response (MSR)	Multi-pathway broad-spectrum off-target effects; significant hepatic and biliary metabolic burden with long-term administration	Tier III	Disappointing Clinical	Very low	Completion of Phase II clinical trials

### 3.1 Antioxidants

Mitochondria-targeted antioxidants (MTAs) represent the most classic strategy for targeting mitochondrial dysfunction. Based on their targeting mechanisms and sites of action, the most extensively studied MTAs currently include triphenylphosphonium (TPP^+^)-conjugated antioxidants represented by MitoQ and SkQ1, as well as cardiolipin-binding membrane stabilizers represented by SS-31. Although these compounds are all dedicated to reducing mitochondrial oxidative stress, their mechanisms of action, targeting sites, and clinical translation pathways differ fundamentally.

MitoQ: MitoQ covalently links the ubiquinone antioxidant scaffold with the triphenylphosphonium (TPP^+^) cation, enabling mitochondrial high enrichment via the negative potential of the mitochondrial inner membrane, thereby neutralizing superoxide anions *in situ* and alleviating lipid peroxidation damage (Young and Franklin, 2019). Studies have shown that adding MitoQ to drinking water daily can prevent cognitive decline and Alzheimer’s-like pathology in mice carrying mutant human transgenes that cause early-onset AD ((Pszczołowska et al., 2024)). Its therapeutic efficacy is highly dependent on the integrity of the membrane potential, and its effect is significantly reduced when the membrane potential collapses in late-stage AD neurons. Animal experiments demonstrate that long-term oral administration of MitoQ can inhibit amyloid plaques, tau hyperphosphorylation, gliosis, and synapse loss in 3xTg-AD mice, regardless of whether intervention is initiated before disease onset at 2 months of age or after pathological damage has occurred at 12 months of age, and it also prolongs the lifespan of the model animals (Young and Franklin, 2019).

Although MitoQ has demonstrated remarkable cognitive improvement in 3xTg-AD mice, its path to clinical translation has not been straightforward. In a Phase II randomized double-blind placebo-controlled clinical trial for Parkinson’s disease (PD), MitoQ (40 mg/day or 80 mg/day for 12 months) was safe and well-tolerated but failed to significantly improve the Unified Parkinson’s Disease Rating Scale (UPDRS) scores for activities of daily living or cognitive function in PD patients (Snow et al., 2010). This negative result suggests that the clinical efficacy of MitoQ may be far less significant than that implied by animal experiments.

Furthermore, the safety of MitoQ also needs to be carefully evaluated. The efficacy of MitoQ is highly dependent on the integrity of the mitochondrial membrane potential. It should be noted, however, that at supra-therapeutic concentrations in certain experimental systems, the intramolecular ubiquinone skeleton has been reported to undergo redox cycling, potentially leading to excessive ROS generation and pro-oxidant effects. Additionally, the TPP^+^ moiety lacks neuronal specificity and distributes to mitochondria in peripheral tissues, raising theoretical concerns about potential disruption of normal redox signaling, although long-term safety data in humans remain limited. These observations suggest that the therapeutic window may be relatively narrow, and this should be carefully considered in future clinical trial designs for elderly AD populations. (Rodriguez-Cuenca et al., 2010). Despite no serious adverse events were reported in Phase II clinical trials of PD, safety data on long-term, high-dose use remain limited (Snow et al., 2010). Critical note: The MitoQ story epitomizes the translational challenges facing mitochondrial-targeted therapies. Despite robust and reproducible efficacy across multiple transgenic AD mouse models (3xTg-AD, APP/PS1) with different intervention windows, the negative Phase II trial in Parkinson’s disease demands careful interpretation. Several factors may explain this disconnect: (i) the inherent limitations of transgenic mouse models, which overexpress familial AD mutations and do not fully recapitulate the multifactorial etiology of sporadic AD—the predominant form of the disease; (ii) the possibility that once clinical symptoms manifest, mitochondrial damage has already progressed to an irreversible stage, suggesting that the therapeutic window for mitochondrial antioxidants may be considerably earlier than currently pursued in trials; (iii) the narrow therapeutic index of TPP^+^-conjugated compounds, where the dose required for central nervous system efficacy may approach or exceed the threshold for pro-oxidant toxicity in peripheral tissues; and (iv) the absence of biomarkers for patient stratification, which may have resulted in enrollment of patients unlikely to respond due to genetic heterogeneity in mitochondrial function (e.g., mtDNA haplogroup backgrounds). These cautionary lessons should inform future trial designs for AD, emphasizing the need for earlier intervention, biomarker-guided patient selection, and combination regimens that address multiple mitochondrial defects simultaneously.

SS-31 (Elamipretide): SS-31 is a tetrapeptide structure with a targeting mechanism that is completely distinct from TPP^+^-class drugs: it specifically binds to cardiolipin, a unique component of the inner mitochondrial membrane, and this binding process does not depend on membrane potential, making it more suitable for late-stage lesions with severe mitochondrial damage. By preserving the structural tethering between endoplasmic reticulum and mitochondria at MAM contact sites, SS-31 restrains excessive ER-to-mitochondria calcium flux and prevents pathological mitochondrial calcium overload; meanwhile, its protection against oxidative protein damage alleviates impaired mitochondrial unfolded protein response (UPRmt) and restores mitochondrial proteostasis.

In addition to scavenging ROS, SS-31 can stabilize inner mitochondrial membrane structure, block abnormal opening of mitochondrial permeability transition pores (mPTP), and inhibit mitochondrial swelling and apoptosis (Qian et al., 2025) (Jia et al., 2023b). ROS-responsive nanomicelles CsA-TK-SS-31 loaded with SS-31 can penetrate the blood-brain barrier and release drugs only in the high oxidative environment of damaged neurons and microglia, simultaneously balancing fusion and division, and alleviating neuroinflammation in 5×FAD mice (Qian et al., 2025). Continuous intraperitoneal administration for 8 weeks to 8-month-old SAMP8 mice can repair hippocampal mitochondrial vacuolization and reverse learning and memory deficits (Huo et al., 2022). In addition to scavenging ROS, SS-31 can stabilize inner mitochondrial membrane structure, block abnormal opening of mitochondrial permeability transition pores (mPTP), and inhibit mitochondrial swelling and apoptosis.

However, long-term administration is prone to accumulation in the liver and adipose tissues, and there is insufficient evidence from human clinical trials. Although SS-31 is not driven by membrane potential, its binding to cardiolipin requires that cardiolipin be maintained in a normal redox state. In late-stage AD patients, the degree of peroxidation of cardiolipin within mitochondria is significantly elevated. This not only reduces drug binding efficiency, but excessive SS-31 also binds to normal cardiolipin in peripheral cells, disrupting mitochondrial homeostasis in hepatocytes and adipocytes, resulting in obvious off-target organ toxicity.

SKQ1: SkQ1 is also equipped with TPP^+^ cations that enrich via membrane potential, but its antioxidant scaffold is plastoquinone, differing from the ubiquinone structure of MitoQ. It suggests notable efficacy in the natural aging OXYS rat model. Long-term oral administration can alleviate hippocampal neuron loss and synaptic damage, downregulate Aβ_1-42_ and tau phosphorylation levels, and simultaneously improve multiple systemic aging phenotypes (Stefanova et al., 2019; Kolosova et al., 2017).

It is worth noting that although SkQ1 has shown significant efficacy in the OXYS rat model, its therapeutic window is relatively narrow. Following long-term high-dose administration, its quinone structure may also induce adverse pro-oxidative side effects. Additionally, it relies on TPP^+^ for non-specific targeting of whole-cell organelles, leading to widespread off-target perturbations in peripheral somatic cell mitochondria. These factors, to some extent, limit its clinical application potential.

Cerium Dioxide Nanoparticles (CeNPs): Cerium dioxide nanoparticles do not belong to small-molecule TPP^+^ or peptide antioxidant systems. Their core characteristic is the dual mimetic enzyme activity of superoxide dismutase (SOD) and catalase (CAT), which is achieved through reversible redox cycling of Ce^3+^/Ce^4+^ on the particle surface, enabling long-term catalytic activity without consumption of the nanoparticle itself. Ce^4+^ captures superoxide anions and reduces them to Ce^3+^, converting O_2_^−^ into oxygen; Ce^3+^, after binding with H_2_O_2_, is reoxidized to regenerate Ce^4+^, continuously decomposing free radicals. T-CeNP modified with receptor for advanced glycation end products (RAGE)-targeting peptides can efficiently penetrate the blood-brain barrier. On one hand, it inhibits the NF-κB pathway to reduce microglial M1 polarization; on the other hand, it blocks Aβ fibrillation while enhancing microglial phagocytosis and degradation of Aβ. It intervenes in AD pathology through multiple pathways including oxidative stress, neuroinflammation, and amyloid deposition (Liu et al., 2025).

### 3.2 Structural stabilizers: rebuilding the scaffold

Drp1 is the core executor of mitochondrial fission and a key target for AD treatment. Inhibiting its excessive activity can correct kinetic imbalance.

Mdivi-1: It is a small molecule Drp1 inhibitor that blocks mitochondrial fission by inhibiting the GTPase activity of Drp1. Studies have shown that inhibiting Drp1 can improve synaptic function, reduce Aβ deposition, and ameliorate cognitive impairment (Dhapola et al., 2022).

P110: Another Drp1 inhibitor works by specifically blocking the interaction between Drp1 and Fis1, a receptor on the mitochondrial membrane, thereby preventing the recruitment of Drp1 to the mitochondria. This mechanism suppresses mitochondrial division, reduces reactive oxygen species (ROS) production, and inhibits apoptosis.

Mdivi-1 and P110 are both Drp1-specific inhibitors that can alleviate excessive mitochondrial fragmentation under AD pathological conditions; however, both have a narrow therapeutic index and prominent off-target effects. Long-term drug administration non-specifically inhibits mitochondrial fission function in normal neurons, glial cells, and peripheral organs, disrupting the basal mitochondrial quality control system. Only low-dose intervention in the early stages of disease is beneficial. Long-term or high-dose use can disrupt the normal mitochondrial remodeling process, exacerbate cellular metabolic damage, and thus limit clinical application.

### 3.3 Quality control enhancers: clearing and replenishing

#### 3.3.1 Mitophagy inducers

Spautin-1: Mechanistically, Spautin-1 was initially characterized as an inhibitor of the deubiquitinating enzymes USP10 and USP13, leading to the suppression of macroautophagy (Liu et al., 2011). However, a recent study identified an unexpected, dual function: Spautin-1 can also promote PINK1-PRKN-dependent mitophagy. The proposed mechanism involves the binding of Spautin-1 to the mitochondrial outer membrane translocase TOMM70, which prevents the import and subsequent degradation of PINK1, leading to its stabilization on the mitochondrial outer membrane and the initiation of mitophagy (Yi et al., 2024). It is important to note that the TOMM70 binding mechanism remains a recent hypothesis requiring further validation, and the classical USP10/USP13 inhibition pathway may also contribute to the molecule’s complex pharmacological effects. The molecular pathways of Spautin-1 are extensive, leading to off-target interference with the ubiquitin-proteasome system and lysosomal maturation processes in addition to targeting specific sites, resulting in a narrow therapeutic window.

Y040-7,904: A potential mitochondrial autophagy regulator, Y040-7,904, was screened out using an artificial intelligence-assisted fluorescence microscopy system (AI-FM). This substance enhances mitophagy by promoting the transport of mitochondria to autophagosomes and the fusion of autophagosomes with autolysosomes. Y040-7,904 also reduces β-amyloid pathology in both *in vitro* and *in vivo* models of AD ((Wang et al., 2025b)).

Rapamycin: A single-center, open-label Phase I clinical trial evaluated rapamycin in mild-to-moderate AD patients. The results showed that no parent drug components were detected in the cerebrospinal fluid (CSF) of subjects following rapamycin intervention. However, AD-related biomarkers in CSF, including phosphorylated tau protein (p-tau), glial fibrillary acidic protein (GFAP), and neurofilament light chain, as well as multiple inflammatory markers in plasma, were significantly elevated (Gonzales et al., 2025). These CSF biomarker changes are concerning: p-tau elevation is generally considered a hallmark of AD pathology progression, and GFAP elevation indicates astrocytic activation and neuroinflammation. In the context of AD clinical trials, such changes are typically interpreted as adverse signals rather than evidence of therapeutic efficacy. The original study suggested that the subtherapeutic drug exposure in the central nervous system, rapamycin was not detected in the CSF, may have been insufficient to achieve therapeutic effects and could potentially account for these paradoxical biomarker findings. Taken together, these data do not support a net beneficial effect of rapamycin on AD-related pathology at the dose and duration tested, and underscore the need for careful monitoring of these biomarker signals in future trials. Microglia-Liposome Fusion Extrusion (MiLi-FE) is a method for preparing microglia-derived nanovesicles (AR@ENV), with which co-delivery of AR7 (a CMA inducer) and rapamycin (an autophagy inducer) can be achieved. It is able to effectively cross the blood-brain barrier. Once inside the cells, they simultaneously activate two autophagy pathways: AR7 antagonizes the retinoic acid receptor alpha (RARα) to enhance chaperone-mediated autophagy, while rapamycin inhibits mTOR to promote macroautophagy. This synergistic activation enhances the clearance of beta-amyloid (Aβ) and other toxic protein aggregates, restores protein homeostasis, and provides neuroprotective effects. Furthermore, this strategy also ameliorates neuroinflammation and significantly rescues cognitive deficits in APP/PS1 mouse model of AD ((Li et al., 2025)).

Urolithin A (UA): Urolithin A improves Alzheimer’s disease cognition and restores mitophagy and lysosomal functions. UA can also activate other pathways independent of PINK1/Parkin, ultimately leading to the degradation of defective mitochondria. Long-term treatment with UA can normalize the abnormal lysosomal cathepsin (primarily cathepsin Z, CTSZ) in the brains of AD mice (including APP/PS1, 3xTgAD, and 3xTgAD/Polβ+/−), thereby restoring lysosomal function (Ho et al., 2024). Lysosomes are the terminal stations of the autophagy process, and the restoration of their function indicates that the entire autophagy flux is unimpeded, which is a key molecular mechanism through which UA exerts its therapeutic effects.

There is significant inter-individual variation in population metabolism of UA, leading to marked fluctuations in the effective drug concentration within the body. Long-term high-dose interventions can excessively activate mitochondrial autophagy in intestinal epithelial cells, leading to off-target mitochondrial damage in the gastrointestinal tract, and long-term organ toxicity data in humans remain lacking.

Kaempferol and dinatyetinicin effectively induce mitophagy in cell, nematode and mouse models, significantly inhibits Aβ and tau pathology, and improves learning and memory abilities in model animals (Xie et al., 2022).

#### 3.3.2 Biogenesis activators (PGC-1α activator and sirtuin modulator)

Mitochondrial biogenesis is a coordinated transcriptional program responsible for synthesizing intact functional mitochondria to replace damaged organelles. Peroxisome proliferator-activated receptor gamma coactivator 1-alpha (PGC-1α) serves as the central transcriptional hub regulating this process, while multiple upstream and parallel regulatory proteins, including AMPK, SIRT1, SIRT3, SIRT6, and ERRα, collectively form a multi-level signaling network that synergistically maintains mitochondrial mass and respiratory function under both physiological conditions and Alzheimer’s disease pathological environments.

SIRT1 is an NAD^+^-dependent nuclear deacetylase and a major upstream regulator of PGC-1α. SIRT1 is believed to activate PGC-1α through deacetylation, a modification that appears to stabilize its protein structure and enhance its transcriptional activity; activated PGC-1α in turn upregulates nuclear respiratory factors 1/2 (NRF1, NRF2), leading to increased expression of TFAM. TFAM, once transported into mitochondria, promotes mtDNA replication and the transcription of electron transport chain subunits—a pathway that, when enhanced, may help restore cellular energy supply under experimental conditions (Yin et al., 2022) (Gong et al., 2013). In the brains of AD patients, SIRT1 expression is reduced, leading to increased acetylation levels of PGC-1α and decreased transcriptional activity, which in turn results in reduced mtDNA copy number and declined mitochondrial quality. Multiple studies have confirmed the therapeutic potential of activating the SIRT1/PGC-1α pathway.

Rhein: Rhein can upregulate downstream NRF-1 and promote mitochondrial biogenesis by activating the SIRT1/PGC-1α pathway in the APP/PS1 transgenic AD mouse model. It not only enhances SOD activity to scavenge ROS, but also repairs mitochondrial function and reduces ROS production by promoting mitochondrial fusion, while alleviating cerebral Aβ deposition, neuroinflammation, and neuronal apoptosis (Yin et al., 2022). However, at high concentrations, it off-targetly inhibits mitochondrial respiratory chain complexes in the kidneys and liver, resulting in significant organ toxicity. This makes it unsuitable for long-term continuous intervention in AD, as its effective therapeutic window is relatively narrow.

Nicotinamide riboside (NR): As a precursor of NAD^+^, effectively increases intracellular NAD^+^ levels, activates SIRT1, upregulates PGC-1α, ultimately promotes mitochondrial gene expression and accelerates the degradation of β-secretase (BACE1), thereby restoring cognitive function in the Tg2576 AD mouse model (Gong et al., 2013). However, Long-term elevation of cellular NAD^+^ levels non-selectively activates sirtuins in peripheral tissues, resulting in metabolic remodeling in the liver and adipose tissue. The safe therapeutic window for long-term drug administration in the elderly population remains undefined. Supraphysiological doses may accelerate the accumulation of somatic mtDNA mutations, posing potential long-term risks.

24-hydroxycholesterol::24-Hydroxycholesterol enhances the expression of PGC-1α and Nrf2 by upregulating SIRT1, triggering the ubiquitin-proteasome degradation of Tau protein, and counteracting the Tau pathology of AD through the SIRT1/PGC-1α/Nrf2 pathway (Testa et al., 2023).

Resveratrol: The classic SIRT1 activators such as resveratrol in regulating Aβ metabolism, Tau protein phosphorylation, and neuroinflammation have been extensively studied, and their mechanisms are closely related to the activation of SIRT1(55). However, A 12-month double-blind, placebo-controlled trial (n = 119) evaluated the effects of resveratrol (500–1,000 mg/day, with daily escalation) on patients with mild to moderate AD. The results reported that resveratrol stabilized Aβ40 levels in CSF and improved brain volume changes (assessed by MRI), but did not show statistically significant improvements in primary cognitive endpoints (ADAS-Cog and MMSE) (Turner et al., 2015). In addition to inconsistent efficacy in clinical trials, resveratrol has a broad target profile and can off-target act on multiple kinases and histone modification pathways throughout the body. Extremely low oral bioavailability necessitates high-dose administration in clinical practice, significantly increasing gastrointestinal adverse reactions and hepatic metabolic burden, with a narrow therapeutic-to-toxic dose interval. Critical note: Resveratrol exemplifies the challenges of translating pleiotropic natural compounds into Alzheimer’s disease therapeutics. Its broad target profile, which includes multiple kinases, sirtuins, and inflammatory pathways, renders it pharmacologically dirty, with off-target effects that are difficult to predict or monitor. Moreover, its extremely low oral bioavailability, less than 1 percent, necessitates high-dose administration, which increases the risk of gastrointestinal and hepatic adverse effects without ensuring sufficient brain penetration. The inconsistent clinical outcomes, such as positive biomarker signals but negative cognitive endpoints, highlight a fundamental dilemma. It remains unclear whether resveratrol truly engages relevant targets in the human AD brain at achievable concentrations, and whether those targets are sufficiently disease-modifying. Until these questions are addressed with improved formulations, for example, nanotechnology-based delivery, and biomarker-guided patient selection, resveratrol should be regarded as a promising but unproven candidate requiring further rigorous evaluation. Furthermore, the discrepancy between its robust mechanistic effects in cell culture, often at micromolar concentrations, and its poor pharmacokinetic profile in humans, with nanomolar plasma levels, raises serious concerns about the translational relevance of many *in vitro* studies. This underscores the critical need for more physiologically relevant experimental models and better bioavailability data in preclinical screening.

AMP-activated protein kinase (AMPK) is a major sensor of cellular energy status and is activated under conditions of energy depletion (low ATP/high AMP). AMPK enhances the transcriptional activity of PGC-1α through direct phosphorylation, while also indirectly activating SIRT1 by increasing NAD^+^ levels, thereby initiating the mitochondrial biogenesis program (Hardie et al., 2012). In AD, AMPK activity dysregulation manifests as a bidirectional abnormality: it may be compensatorily elevated in early stages and significantly reduced in late stages. AMPK activators such as metformin show potential in AD models to promote mitochondrial biogenesis, improve energy metabolism, and cognitive function (Wang et al., 2019).

Unlike SIRT1, which is localized in the nucleus, SIRT3 is a deacetylase primarily localized in the mitochondrial matrix, directly regulating the activity of mitochondrial metabolic enzymes. SIRT3 activates multiple metabolic enzymes, such as superoxide dismutase 2 (SOD2), isocitrate dehydrogenase 2 (IDH2), and long-chain acyl-CoA dehydrogenase (LCAD), through deacetylation, thereby enhancing mitochondrial antioxidant defense and fatty acid oxidation capacity (Ansari et al., 2017). Importantly, SIRT3 forms a positive feedback loop with PGC-1α. PGC-1α promotes SIRT3 transcription, while SIRT3 maintains cellular NAD^+^/NADH homeostasis by enhancing mitochondrial function, indirectly supporting SIRT1 activity. Curcumin, which has neuroprotective effects, alleviates Aβ-induced neuronal metabolic dysfunction and improves cognitive performance in a mouse model of AD by increasing SIRT3 activity (Liu M. et al., 2021).

SIRT6 is another important NAD^+^-dependent deacetylase, primarily localized in the nucleus, involved in regulating DNA repair, telomere maintenance, and glucose metabolism. Recent studies have revealed that SIRT6 can regulate mitochondrial biogenesis through interactions with PGC-1α and NRF1((Chang et al., 2020)). Depletion of SIRT6 in the brain occurring during aging and AD leads to increased GSK3 activity, phosphorylated Tau, neurodegeneration caused by DNA damage, and behavioral deficits (Kaluski et al., 2017). In the pathological context of AD, decreased SIRT6 expression may impair both DNA repair capacity and mitochondrial function.

Estrogen-related receptor alpha (ERRα) is an orphan nuclear receptor that forms a bidirectional regulatory axis with PGC-1α. ERRα is an effector molecule of the transcriptional coactivator PGC-1α and regulates the expression of genes involved in oxidative phosphorylation and mitochondrial biogenesis. Inhibition of ERRα impairs the ability of PGC-1α to induce the expression of mitochondrial protein-coding genes and increase mitochondrial DNA content (Schreiber et al., 2004). In AD, changes in ERRα expression levels are closely associated with energy metabolism disorders.

In summary, although the SIRT1/PGC-1α pathway is a classic core axis regulating mitochondrial biogenesis, it does not operate in isolation. AMPK functions as an energy sensor to initiate biogenic signals; SIRT3 and SIRT6 maintain mitochondrial function at the mitochondrial and nuclear genome levels, respectively; ERRα acts as a key coactivator of PGC-1α, amplifying its transcriptional effects. These regulatory factors are interconnected and mutually complementary, collectively maintaining the health and dynamic balance of the mitochondrial network. In AD treatment strategies, solely targeting SIRT1/PGC-1α may not be sufficient to fully restore mitochondrial function, necessitating multi-level interventions that simultaneously consider activating AMPK, restoring SIRT3 and SIRT6 levels, and enhancing ERRα activity.

Currently, multiple active ingredients of traditional Chinese medicine have been confirmed to act on this multi-level regulatory network. Schisandrin B, isoliquiritigenin, berberine, and Lycium barbarum polysaccharides, can improve disease conditions by activating the mitochondrial quality control (MQC) system, which includes promoting mitochondrial biogenesis (Zhang et al., 2025).

### 3.4 Coenzyme Q10

Coenzyme Q10 (CoQ10) is an endogenous lipophilic cofactor embedded within the inner mitochondrial membrane, serving as a key intermediate in the, ETC., and possessing inherent antioxidant activity (Fišar and Hroudová, 2024). Accompanying aging and the pathological progression of AD, the content of coenzyme Q10 in brain tissue continuously decreases, exacerbating electron leakage and the mass production of ROS, thereby forming mitochondrial oxidative damage. Exogenous supplementation with coenzyme Q10 can alleviate respiratory chain blockage, stabilize mitochondrial membrane potential, and restore energy supply to neurons. Coenzyme Q10 and its analogs (such as the mitochondrial-targeted antioxidant MitoQ) have shown potential in preclinical studies to enhance mitochondrial function, reduce oxidative stress, and improve cognitive abilities in animal models (D'Alessandro et al., 2025).

Clinical research results of CoQ10 in the field of AD are generally disappointing. Although CoQ10 successfully reduced oxidative damage markers (F2-isoprostanes) in the CSF of AD patients, indicating that it indeed exerted antioxidant functions at its target site (mitochondria) *in vivo*, this biochemical improvement did not translate into clinical cognitive improvement (Galasko et al., 2012). In PD, a multicenter placebo-controlled trial (QE2 study) involving 80 PD patients showed that CoQ10 (1,200 mg/day for 16 months) failed to delay disease progression in early PD patients (no significant improvement in UPDRS scores) (Beal et al., 2014). These studies confirm that supplementing with CoQ10 alone is insufficient to reverse established mitochondrial damage.

Coenzyme Q10 presents a significant transformation bottleneck. Oral bioavailability is extremely low. And exogenous high-dose supplementation of coenzyme Q10 disrupts the endogenous respiratory cofactor homeostasis in non-lesional tissues, leading to systemic redox imbalance and off-target effects. Its lipophilic structure results in significant accumulation in adipose tissue, resulting in a narrow therapeutic window between effective and metabolically disruptive doses, thereby limiting its value as a monotherapy for late-stage AD. Critical note: The CoQ10 story exemplifies a recurring challenge in mitochondrial medicine—the dissociation between target engagement and clinical efficacy. A randomized controlled trial in AD patients demonstrated that CoQ10 supplementation successfully reduced cerebrospinal fluid F2-isoprostanes (a marker of oxidative damage), confirming that the drug reached its intended target and exerted its biochemical effect. However, this biomarker improvement did not translate into cognitive benefit. Similarly, the QE2 trial in Parkinson’s disease (n = 80, 1,200 mg/day for 16 months) showed no significant slowing of disease progression. These negative findings suggest that: (a) oxidative stress is a downstream consequence rather than a root cause of the neurodegenerative cascade; (b) late-stage interventions that do not address upstream drivers such as impaired mitophagy or mtDNA damage are unlikely to yield clinical benefit; and (c) the therapeutic window for mitochondrial antioxidants may be considerably narrower than preclinical studies suggest, with high doses potentially disrupting endogenous redox homeostasis in non-target tissues.

### 3.5 Curcumin

Curcumin, the primary active component of turmeric, regulates mitochondrial stress homeostasis through the JMJD3-H3K27me3 signaling axis and exerts neuroprotective effects via multiple pathways. It can directly combine with Aβ monomers and oligomers, block amyloid fiber formation, while inhibiting abnormal binding of Aβ to mitochondrial functional proteins, thereby alleviating mitochondrial structural damage and reducing neuronal oxidative apoptosis. Curcumin intervention can improve the brain tissue morphology and pathological changes, and inhibit neuronal apoptosis in AD model mice. In behavioral tests (such as the Morris water maze), curcumin can improve the spatial learning and memory ability of APP/PS-1 double-transgenic mouse models of AD ((Li J. et al., 2021)). This is closely related to its comprehensive effects of alleviating oxidative stress, inhibiting inflammation, and protecting mitochondrial function.

Although curcumin has demonstrated extensive neurotrophic and neuroprotective effects in animal models of AD, its performance in clinical studies has also been inconsistent. A 12-month randomized, double-blind, placebo-controlled trial (n = 36) showed no significant difference in cognitive scores (ADAS-Cog) between the curcumin treatment group and the placebo group (Baum et al., 2008). Another clinical trial of a high-bioavailability curcumin formulation (Theracurmin) observed a small improvement in Mini-mental State Examination (MMSE) scores compared to the placebo group after 18 months of treatment, but it did not reach the prespecified statistical significance (Sma et al., 2018).

The primary obstacle to the clinical translation of Curcumin is extremely low bioavailability. After oral administration, it is rapidly metabolized via the gastrointestinal tract, undergoes extensive first-pass hepatic clearance, resulting in an extremely short half-life in the body and insufficient effective drug concentrations in brain tissue. On the other hand, Curcumin non-specifically targets multiple intracellular pathways making off-target side effects difficult to predict with long-term administration.

Critical appraisal of the evidence base: The therapeutic strategies summarized above differ substantially in their evidentiary robustness. I categorize the available evidence into four tiers (Tier I) Mechanistically well-established with consistent replication across ≥3 independent laboratories and ≥2 animal models (e.g., MitoQ in 3xTg-AD and APP/PS1 mice; SS-31 in SAMP8 and 5×FAD mice) (Tier II) Supported by single high-quality studies but awaiting independent replication (e.g., Spautin-1-mediated mitophagy via TOMM70 binding; Y040-7,904 identified by AI-assisted screening) (Tier III) Preclinically promising but associated with significant translational failures or conflicting clinical data (e.g., CoQ10; resveratrol) (Tier IV) Preliminary observations requiring validation in more physiologically relevant models (e.g., certain herbal compounds with undefined active constituents or unclear mechanisms). Evidence Priority classification: Strong = Tier I multi-model consistent preclinical efficacy; Moderate = Tier II single high-quality animal experiment; Preliminary = Tier II only cell/*in vitro* data without *in vivo* validation; Disappointing Clinical = Tier III with failed Phase II/III clinical trials. Readers should interpret the therapeutic potential of each agent within this evidence hierarchy, recognizing that the gap between robust preclinical findings and clinical benefit remains substantial—a challenge shared across the AD drug development field.

## 4 A multidimensional synergistic therapeutic strategy for MER

Although mitochondrial-targeted therapies have demonstrated significant potential in AD research, the complexity of the AD pathological network dictates that single-target interventions often prove ineffective. The traditional ‘single drug-single target’ paradigm is ill-equipped to address the vicious cycle characterized by intertwined oxidative stress, dysregulated dynamics, impaired quality control, and neuroinflammation in mitochondrial dysfunction.

### 4.1 Synergistic mitochondrial function restoration and aβ-targeted clearance

In AD brains, Aβ plaques not only deposit extracellularly but also specifically accumulate on mitochondrial membranes through mechanisms such as the ABAD receptor, directly blocking the electron transport chain and triggering oxidative bursts. Simple Aβ clearance, such as monoclonal antibodies, may release a large amount of soluble oligomers, leading to inflammatory rebound and secondary mitochondrial damage. Therefore, the combined strategy of ‘Aβ clearance and mitochondrial repair’ is crucial. Pre-arm neurons with mitochondrial-targeted antioxidants (e.g., MitoQ) or membrane-stabilizing peptides (e.g., SS-31) to neutralize ROS within mitochondria and stabilize mitochondrial membrane potential (D'Alessandro et al., 2025). On this basis, combine Aβ immunotherapy or BACE1 inhibitors, which can eliminate the pathological source while protecting neurons from damage caused by toxic substances released during Aβ clearance and off-target effects. The nano-drug CsA-TK-SS-31 (CTS) achieves the simultaneous release of cyclosporine A (CsA) and SS-31 in response to the high ROS environment within mitochondria, thereby inhibiting mPTP opening while mitigating oxidative damage (Qian et al., 2025). This provides an example of a combined therapeutic strategy that addresses both intracellular and extracellular aspects.

### 4.2 Mitophagy inducers and tau pathology intervention

Impaired mitophagy results in the accumulation of dysfunctional mitochondria, which serves as a key driver of energy crisis and neuroinflammation in AD. However, the restoration of mitophagy alone may be insufficient to address coexisting tau protein pathology. Co-administration of mitophagy inducers (e.g., Urolithin A or Spautin-1) with Tau-targeted drugs (e.g., Tau aggregation inhibitors or kinase inhibitors), aiming to simultaneously initiate clearance of damaged mitochondria and Tau tangles. Enhancing mitochondrial autophagy can inhibit the aggregation of Aβ and Tau proteins and improve cognition (Xie et al., 2022). By restoring the PINK1/Parkin pathway, not only are damaged mitochondria cleared, but tau hyperphosphorylation induced by mitochondrial dysfunction is also indirectly reduced. Such combination therapies demonstrate significant superiority over monotherapy in improving cognitive function, reflecting a systematic remodeling of the intracellular homeostatic environment.

### 4.3 Comprehensive regulation of the metabolic-immune axis

Mitochondria function not only as energy factories but also as immune regulators. In AD, damaged mitochondria release mtDNA as DAMPs, which drive microglia toward a pro-inflammatory (M1) phenotype via pathways such as cGAS-STING, thereby establishing a vicious cycle of ‘metabolic damage → inflammation → neurodegeneration’. Effective treatment must disrupt this cycle. Restoration of mitochondrial function itself constitutes an anti-inflammatory strategy. Co-administration of PGC-1α activators with specific anti-inflammatory drugs.

Many traditional Chinese medicine (TCM) formulas (e.g., Huanglian Jiedu Decoction) or individual compounds (e.g., berberine, icariin) inherently possess multi-target properties. Berberine improves AD-related mitochondrial damage through the AMPK/SIRT1 and PINK1/Parkin dual pathways. In an *in vitro* neuronal model of Aβ oligomers, berberine significantly reduces intracellular ROS accumulation, stabilizes mitochondrial membrane potential, and simultaneously inhibits NLRP3 inflammasome-mediated neuroinflammation. Behavioral studies in animals confirm that berberine can improve spatial memory deficits in AD mice and reduce Aβ plaque deposition in the hippocampus (Zhang et al., 2025). In SAMP8 aging AD model mice, intervention with icariin significantly increased mtDNA copy number, inhibited Drp1 overexpression, and alleviated neuronal apoptosis induced by tau hyperphosphorylation; simultaneously, icariin can block the cGAS-STING pathway activated by mtDNA release, thereby reducing microglial M1 polarization and chronic neuroinflammation (Zhang et al., 2025). Unlike single entities, traditional Chinese medicine compound formulas rely on the synergistic action of multiple components to exert their effects. Huanglian Jiedu Decoction contains active components such as berberine, baicalin and geniposide, synchronously intervening in the three mitochondrial pathological processes of AD: clear excessive reactive oxygen species, balance mitochondrial fission and fusion, and enhance mitochondrial autophagic flux. *In vivo* experiments confirmed that this compound formula downregulates the activity of the NF-κB and NLRP3 pathways, reduces Aβ deposition in the brain, and improves synaptic loss in 5xFAD mice (Zhang et al., 2025). Compared to single monomers, TCM compound formulas exhibit milder pharmacological effects but exert more comprehensive regulation on the mitochondrial metabolism-immune axis.

### 4.4 The future landscape of rational polypharmacy

**FIGURE 3:**
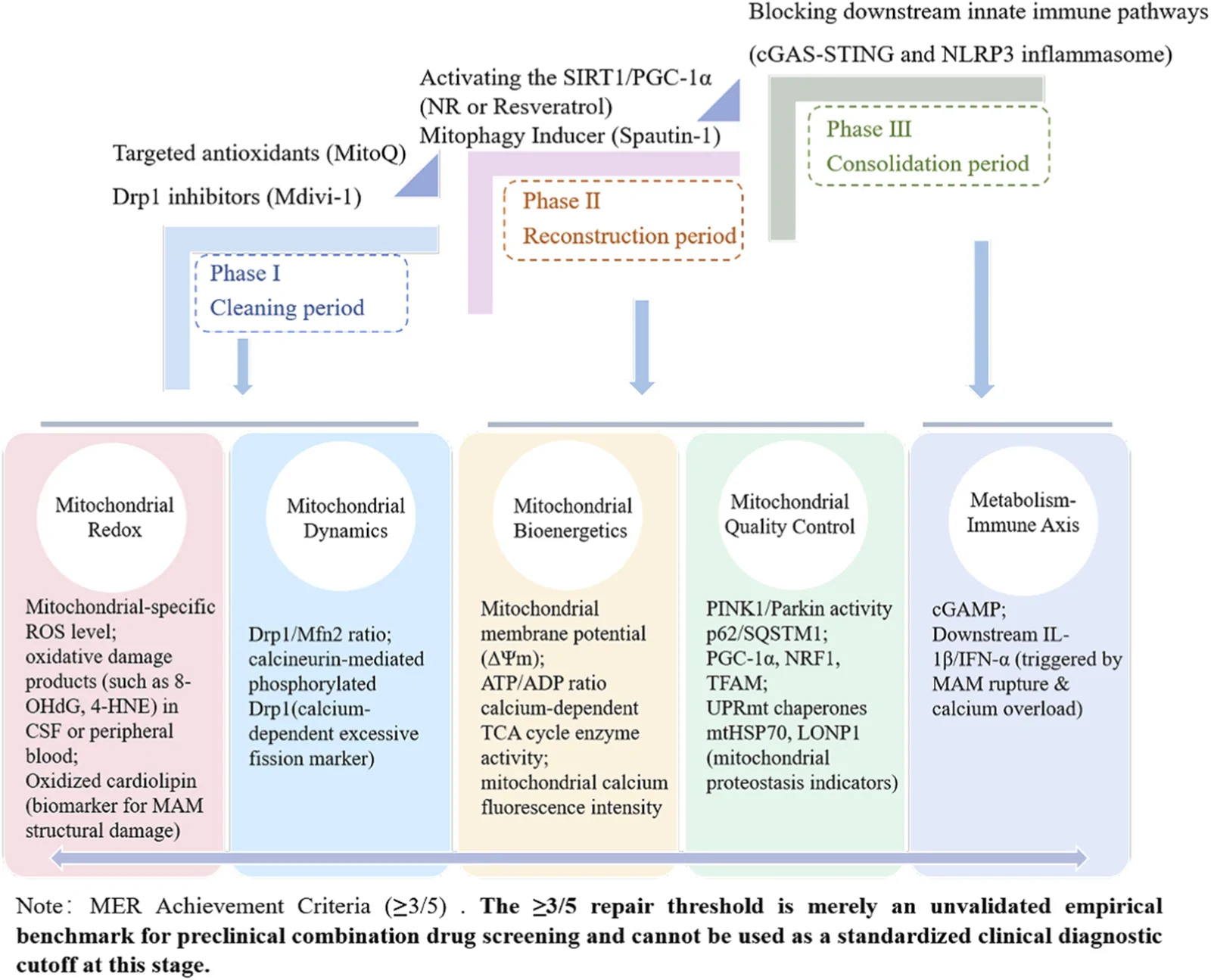
Mitochondrial Ecological Triple Therapy. All drug target pathways, molecular markers and pathological indicators depicted in the figure correspond to mitochondrial dysfunction pathways and biomarkers that have been validated by existing independent preclinical and clinical studies, representing conclusive biological evidence. The colored dashed modules (Phase I, Phase II, and Phase III) and the core assessment criterion of “≥3/5 mitochondrial dimension recovery standards” are the initial provisional multi-stage treatment hypothesis proposed in this review, which has not yet undergone systematic calibration or validation through animal model or human cohort trials. The MER framework proposes a phased, multi-target intervention paradigm to systematically restore mitochondrial ecosystem homeostasis in AD. The strategy comprises three sequential phases: Phase I (Cleaning period) focuses on neutralizing excessive ROS using mitochondria-targeted antioxidants (e.g., MitoQ) and correcting excessive mitochondrial fission using Drp1 inhibitors (e.g., Mdivi-1), thereby creating a stable intracellular environment. Phase II (Reconstruction period) aims to activate endogenous regenerative mechanisms by promoting mitochondrial biogenesis via the SIRT1/PGC-1α signaling axis (e.g., nicotinamide riboside or resveratrol), replenishing the healthy mitochondrial pool. Phase III (Consolidation period) targets the blockade of downstream innate immune pathways (cGAS-STING and NLRP3 inflammasome) triggered by mtDNA release, thereby suppressing chronic neuroinflammation and consolidating microenvironmental homeostasis. Each phase targets a distinct dimension of mitochondrial dysfunction. MER is defined as achieved when at least three of the five indicators are simultaneously normalized after intervention. Each of the five MER biomarkers is linked to measurable readouts reflecting MAM integrity, calcium homeostasis and mitochondrial proteostasis. Critical Note: The ≥3/5 recovery threshold is only an unvalidated heuristic screening benchmark for preclinical combinatorial drug research, and cannot be used as standardized clinical diagnostic cutoffs at present.

Future AD therapies should not be limited to the simple stacking of drugs, but should be rationally designed based on pathological networks. According to the operational definition and diagnostic criteria of MER, I propose that future combination therapies should be designed as a stepwise, multi-target, temporally coordinated “Mitochondrial Ecological Triple Therapy” (Figure 3).

Phase I: Cleaning period. This is the cornerstone of repair. Initially, it is necessary to rapidly neutralize the excessive ROS within mitochondria through targeted antioxidants (such as MitoQ), thereby interrupting the sustained damage caused by oxidative stress to mitochondrial DNA and membrane structure. Additionally, targeting the mitochondrial dynamics abnormalities that occur early in AD, Drp1 inhibitors (such as Mdivi-1) (Baek et al., 2017) are utilized to correct excessive mitochondrial fission and restore the integrity of the mitochondrial network. MAM-stabilizing agents such as SS-31 restore the structural integrity of ER-mitochondrial contact sites, restrain aberrant IP3R-VDAC1-mediated calcium shuttling from ER to mitochondria, and cut down the generation of proteotoxic reactive oxygen species triggered by calcium overload. Relevant experimental detection indicators include IP3R and VDAC1 protein expression levels, oxidized cardiolipin content and mitochondrial calcium fluorescent signals. This step aims to create a relatively stable internal environment for the cell and prevent the further spread of damaged mitochondria.

Phase II: Reconstruction period. After clearing obstacles, the focus of treatment should shift to activating the endogenous regenerative mechanisms of cells. By activating the SIRT1/PGC-1α signaling axis (such as NR or Resveratrol) (Gong et al., 2013; AlHayani et al., 2025), it is not only possible to promote mitochondrial biogenesis and replenish the healthy mitochondrial pool, but also to enhance the mitochondrial unfolded protein response (UPRmt), thereby improving the mitochondrial capacity to cope with proteotoxic stress. UPRmt inducers upregulate mitochondrial chaperones (mtHSP70, LONP1) to rescue disrupted mitochondrial proteostasis, while SIRT1/PGC-1α agonists reconstruct functional MAM complexes and replenish intact mitochondria with balanced calcium handling capacity. The measurable markers include PGC-1α, TFAM, mtHSP70 and LONP1 protein abundance. This stage is critical for restoring the metabolic resilience of neurons.

Phase III: Consolidation period. With a deeper understanding of the mechanism of mtDNA as DAMPs, future interventions must shift from simply antioxidant to the level of immune regulation. I advocate for the localization of mitochondria as the immune-metabolic hub, focus on blocking downstream innate immune pathways (such as cGAS-STING and NLRP3 inflammasome) triggered by mtDNA. Blockers of the mtDNA-triggered cGAS-STING and NLRP3 inflammasome cascades interrupt sustained neuroinflammation, which is the downstream pathological outcome of MAM collapse, excessive calcium efflux and damaged mtDNA release. Representative detection indicators include cytosolic mtDNA copy number, phosphorylated STING, cleaved caspase-1 and pro-inflammatory cytokines IL-1β, IFN-α. By blocking this ‘metabolic-immune’ conversion axis, chronic neuroinflammation triggered by mitochondrial damage can be effectively suppressed, thereby achieving long-term protection of the neural microenvironment.

Theoretically, the specific triple combination of Mdivi-1 + NR/resveratrol + MitoQ can meet three-dimensional standards and reach the threshold for MER determination. Although, as of now, it has not been systematically experimentally tested in AD cellular or animal models, existing literature provides indirect evidence supporting a synergistic effect. This triple matching strategy is logically constructed based on the independent protective effects of each monotherapy, targeting three core defective links of mitochondrial dysfunction (abnormal fission, insufficient biogenesis, excessive oxidative burst) simultaneously to break the self-amplifying vicious cycle in AD. Further cellular co-treatment assays and multi-drug combined animal intervention experiments are required to verify the synergistic efficacy and safe dosage window of this triple therapeutic model in future research.

## 5 Experimental models for mitochondrial-targeted therapies in Alzheimer's disease

### 5.1 Animal models

Genetic models are powerful tools for investigating the causal relationship between specific pathogenic proteins (Aβ/tau) and mitochondrial dysfunction. The 5xFAD model can be utilized to investigate how Aβ oligomers directly induce mitochondrial fission, inhibit respiratory chain function, and generate ROS. The 3xTg-AD model can further explore how tau pathology exacerbates mitochondrial transport impairments and energy crises. They provide a controllable experimental system for dissecting the cascade reaction of “Aβ/tau → mitochondrial damage → neuroinflammation” within a well-defined genetic background. The genetic model is constructed based on a small number of Familial AD (FAD) gene mutations, overlooking age as the primary risk factor and the influence of acquired environmental factors, thus failing to simulate the complex etiological network of Sporadic AD (SAD) (Ong et al., 2025). Most transgenic models typically do not exhibit the widespread neuronal loss characteristic of typical human AD (Table 2).

**TABLE 2:** Animal models for mitochondrial-targeted therapies in Alzheimer’s disease.

Classification	Animal models	Advantages and applicable scenarios	Similarities and differences	(Refs.)
Genetically modified models	5xFAD Transgenic Mice	Currently one of the fastest models of Aβ pathology progression. Extremely suitable for short-term intervention studies, such as rapidly evaluating the clearance effect of drugs on Aβ deposition or their improvement of cognitive function	It belongs to Aβ-dominant transgenic models along with APP/PS1 models, but exhibits earlier and more rapid pathological progression. Compared with the 3xTg-AD model, it does not simulate tau protein pathology	(Sweetat et al., 2023)
	APP/PS1 Transgenic Mice	It can stably simulate characteristics such as amyloid plaque formation and cognitive function decline in AD patients. Widely applied to explore the mechanisms of amyloid plaque formation, neuronal death, and to evaluate various potential therapeutic strategies	Homologous to 5xFAD but differing in the number of mutated genes and the speed of pathological progression	(Misrani et al., 2021)
	3xTg-AD mice	Its greatest advantage lies in its ability to simultaneously simulate two core pathological proteins, Aβ and Tau, with pathological progression exhibiting age dependence (Aβ deposition appearing at 6 months of age and Tau tangles emerging at 12–15 months of age). This makes it an ideal platform for studying the interactions between the two pathologies and for validating combined therapies targeting dual pathology	Unlike single Aβ or Tau models, it provides a more complex pathological environment that is closer to the actual situation of multiple pathologies coexisting in the brains of AD patients	(Young and Franklin, 2019)
Aging-related models	SAMP8 mice OXYS Rat	Spontaneous generation of AD-like pathology without the introduction of exogenous human mutant genes,and is considered to have unique value in modeling the vast majority of SAD. They can more comprehensively reflect the association between aging, the greatest risk factor for AD, and the occurrence and development of the disease	Compared with transgenic models, the onset of its pathological changes occurs relatively later, and there may be certain inter-individual variations. OXYS rats, as a rat model, may complement mouse models in some physiological and behavioral tests	(Ong et al., 2025) (Stefanova et al., 2014)
Humanized gene knock-in model	hAbKI (Humanized Aβ Knock-in) mice	Short modeling cycle and relatively simple operation	Typically focus on inducing a specific pathological process or symptom, rather than simulating the complete natural history of the disease	(Jia et al., 2023a; Baglietto-Vargas et al., 2021)

Aging models (SAMP8, OXYS) focus on simulating age-related systemic and neurological functional decline driven by aging itself as the core risk factor, rather than relying on exogenous gene mutations; instead, it achieves accelerated aging phenotypes through natural senescence or selective breeding. The SAMP8 model is the gold standard for studying the ‘metabolism-immunity’ axis and mitochondrial functional decline with age. However, their limitations are equally pronounced. The genetic basis remains complex and not fully elucidated; pathological manifestations involve multiple systems that may interfere with one other; the typicality of certain core pathological features of AD is insufficient; and practical issues such as high breeding costs, low reproductive capacity, and interspecies differences exist.

hAbKI (humanized Aβ knock-in) mice are not traditional chemically induced models but humanized knock-in models constructed through gene editing technology. The human Aβ coding sequences (including Aβ42 and Aβ40) are inserted into the corresponding positions of the mouse APP gene with CRISPR/Cas9 technology, thereby preserving the regulatory elements and expression pattern of the endogenous APP. Compared to traditional APP transgenic models (such as 5xFAD, APP/PS1), the hAbKI model avoids non-Aβ-related phenotypes resulting from APP overexpression (e.g., accumulation of APP-CTFs, cytotoxicity), enabling a more exclusive investigation of the direct impact of Aβ pathology on mitochondrial function (Baglietto-Vargas et al., 2021). At the pathological level, hAbKI mice recapitulate key early lesions of SAD, including progressive accumulation of Aβ42 in synaptic mitochondria, impaired synaptic oxidative phosphorylation activity, and excessive release of ROS. These changes occur prior to the formation of large senile plaques, making hAbKI mice an ideal tool for studying the upstream mechanisms of mitochondrial damage induced by physiological levels of human Aβ in the pre-plaque early stages of AD (10). This model has a moderate modeling cycle and standardized gene editing operations, making it more valuable for translational research on SAD mitochondrial-related mechanisms compared to overexpression transgenic mice. However, the model is limited to simulate Aβ-related pathology and is unable to spontaneously generate tau protein hyperphosphorylation and neurofibrillary tangles. Consequently, it cannot be used alone for studies on tau and mitochondrial interactions.

### 5.2 Human-derived models

Human-derived models, particularly those based on induced pluripotent stem cells (iPSCs) and three-dimensional brain organoids, represent a profound paradigm shift in AD research. They fundamentally address the core bottleneck of interspecies differences between traditional animal models and humans, providing an unprecedented platform for studying disease mechanisms and drug responses within the human genetic context. Researchers utilized human iPSCs differentiated into cortical neurons and conducted long-term *in vitro* culture to simulate the neuronal aging process. By introducing pathogenic β-amyloid (Aβ42) oligomers into this model system, the key pathological features of AD can be successfully induced. The important value of this model lies in its ability to monitor the direct damage of Aβ42 to mitochondrial function in real time and longitudinally. This model has been used to test the efficacy of various therapeutic compounds (such as memantine, donepezil, etc.). By measuring functional indicators such as long-term potentiation (LTP, an electrophysiological marker that simulates the formation of learning and memory), the efficacy of drugs in rescuing synaptic dysfunction caused by Aβ and mitochondrial damage can be evaluated (Gallo et al., 2024).

The Israel team successfully generated iPSCs in 2012, starting from skin fibroblasts of six individuals, by retroviral transduction of the reprogramming factors OCT4, SOX2, KLF4, and c-MYC. This study confirmed that sporadic AD (SAD2) and familial AD neurons exhibit endocytic system dysfunction at the early stages of pathology: Rab5-positive early endosomes abnormally enlarge, and the acidic microenvironment of endosomes continuously activates β-secretase, leading to extensive β-cleavage of APP. This results in increased secretion of Aβ(1–40), sustained activation of GSK-3β, and excessive phosphorylation of tau protein at the Thr231 site, with concurrent elevation of these three factors. The expression levels of Aβ, activated GSK-3β, and phosphorylated tau are strongly positively correlated. Pharmacological intervention experiments further confirmed that only β-secretase inhibitors can synchronously reverse GSK-3β and tau pathology, while γ-secretase inhibitors have no ameliorative effect. This suggests that the upstream molecule mediating abnormal tau phosphorylation is the APP β-cleavage fragment (β-CTF), rather than mature Aβ oligomers. This study clearly identifies endocytic pathway dysregulation and abnormal APP processing as precursor lesions preceding the formation of large Aβ plaques. However, this work did not detect mitochondrial-related indicators such as mitochondrial morphology, oxidative stress, and respiratory chain activity, and thus cannot directly substantiate mitochondrial temporal damage (Israel et al., 2012).

iPSC cortical neuron models from patients with early-onset AD carrying the PS1-E120 K pathogenic mutation confirm that mitochondrial homeostasis imbalance occurs prior to the emergence of prominent Aβ and tau pathologies. In PS1-E120 K iPSC-derived neurons, mitochondrial fission protein Drp1 expression is significantly upregulated, while inhibitory p-Drp1(Ser637) levels are decreased. Additionally, fusion proteins Mfn1/2 and OPA1 expression is markedly downregulated. Proteins PINK1 and PARKIN related to mitochondrial autophagy are significantly increased (Li et al., 2018).

Under specific conditions, three-dimensional culture of iPSCs can enable them to self-organize into brain organoids with complex spatial structures. Compared to traditional two-dimensional cell cultures, brain organoids can more effectively simulate the cellular composition, spatial arrangement, and intercellular interactions of *in vivo* tissues. Human-derived models circumvent species differences, particularly enabling the recapitulation of human-specific mtDNA haplogroup genetic backgrounds. In contrast, rodents lack homologous mitochondrial haplogroup stratification to humans, which is an important reason why drug efficacy observed in animal experiments often fails to translate to clinical settings (Gallo et al., 2024). Brain organoids constructed from iPSCs further confirm that cells of different mtDNA haplogroups exhibit significant differences in their response to PGC-1α agonists and mitochondrial-targeted antioxidants, making them an ideal tool for individualized drug screening (Tolle et al., 2023).

Particularly the impact of mtDNA haplogroup background on drug response. However, challenges remain: limited maturity of organoids, which hinders the full simulation of adult or aged brains; absence of vascular systems and complete immune microenvironments, and the need for enhanced standardization and reproducibility in cultivation (Krefft et al., 2018).

## 6 Emerging assessment tools and biomarkers

### 6.1 Neuroelectrophysiology

Record local field potentials (LFPs) in brain regions such as the hippocampus, and analyze the oscillatory energy in the theta (4–13 Hz) and gamma (30–80 Hz) frequency bands. AD model neural oscillations are weakened, and extremely low-frequency magnetic field therapy can significantly enhance the energy in these frequency bands, which is synchronized with cognitive improvement (Geng et al., 2025).

### 6.2 Imaging evaluation

As 18 F-FDG PET is used to evaluate brain glucose metabolism, AD mice after PPA nanoparticle treatment showed increased FDG distribution in the brain, indicating recovery of neuronal metabolic function (Zhong et al., 2022).

### 6.3 Mitochondrial function index (MFI)

This is a novel composite indicator that integrates multiple parameters of Peripheral Blood Monocytes (PBMCs), including mitochondrial membrane potential, ROS, mass, and cell apoptosis, through flow cytometry. MFI is significantly reduced in AD patients and is highly correlated with cognitive scores (MMSE/CDR), showing potential as a biomarker for dynamically monitoring the efficacy of mitochondrial-targeted therapies (Hauger et al., 2025).

## 7 Progress in clinical research and trials

In recent years, targeting mitochondria to improve their function, clear damaged mitochondria, or protect their structure has become a highly promising new direction in the treatment of AD. Currently, several mitochondrial-targeted therapies based on different mechanisms of action have entered or are about to enter clinical trial stages.

### 7.1 Oral mitochondrial activator: SUL-23

This is an innovative oral small molecule drug developed based on hibernation mechanisms, designed to support cellular energy metabolism by activating mitochondrial complex I and IV. Its mitochondrial activation based on hibernation mechanisms has shown potential to improve mitochondrial function in animal models. Its Phase I clinical trial (targeting healthy elderly volunteers) was completed in 2025. GEN Pharmaceutical Company and Sulfateq BV of the Netherlands have jointly announced that SUL-23 has good safety and tolerability, and can effectively penetrate the blood-brain barrier, with a cerebrospinal fluid concentration accounting for as high as 74.2%. This single-center, first-in-human trial was a randomized, double-blind, placebo-controlled single ascending dose (SAD) study that enrolled 53 healthy elderly adults divided into three parts. The first part included six cohorts (oral doses of 50, 100, 250, 500, 1,000, and 2000 mg, n = 23). The second part evaluated the pharmacokinetics of a single 1,000 mg oral dose in 10 healthy elderly adults. The third part employed a randomized, single oral dose of 2000 mg, double-treatment, double-period crossover design (n = 20) to assess food effect. The trial results show that oral administration of SUL-23 at single doses of 50–2000 mg exhibits good safety and tolerability, with excellent pharmacokinetic characteristics and high cerebrospinal fluid (CSF) penetration. These data indicate that SUL-23 has the potential to become a therapeutic agent for AD and other neurodegenerative disorders.

### 7.2 VNA-318

It is a ‘Mitophagy +' small molecule developed by Vandria Company. Phase I first-in-human trial (2025) has shown that VNA-318 did not exhibit severe adverse reactions in all dose groups, demonstrating good safety and tolerability. This lays the foundation for its long-term use and subsequent patient trials. VNA-318 has been shown in studies to reduce p-tau (phosphorylated tau protein) and neuroinflammatory markers. A Phase IIa proof-of-concept trial is planned to be initiated in 2026, which will include a patient population with mild to moderate cognitive impairment to further verify its disease-modifying effect on improving cognitive function.

### 7.3 PX578

PX578, a mitochondrial DNA polymerase gamma (POLG) activator, is an innovative drug designed to treat neurodegenerative diseases (such as AD) by enhancing mitochondrial DNA (mtDNA) replication and repair capacity. Pretzel Therapeutics Company announced the initiation of Phase I clinical trials for PX578 in April 2025. The primary objective of the trial is to evaluate the safety and tolerability of the drug in humans, including patients with neurodegenerative diseases such as AD, and to conduct dose escalation studies.

### 7.4 Latrepirdine (Dimebon):Critical Ccses of Ccinical Ttanslation Ffilure

Dimebon was originally developed as an antihistamine and later identified as a mitochondrial membrane potential stabilizer, capable of transiently improving mitochondrial function and inhibiting Aβ-induced neurotoxicity. Early Phase II clinical trials previously reported encouraging results, generating great anticipation. However, the subsequent large-scale, multicenter Phase III clinical trial (CONNECTION trial) clearly showed that Dimebon failed to slow cognitive decline in patients with mild to moderate AD, demonstrating no significant difference in efficacy compared to the placebo group, leading to its research and development plan to be terminated (Bezprozvanny, 2010). This failure tells us positive phase II results do not always predict success in phase III clinical trials; simple stabilization of membrane potential may be insufficient to counteract the complex multidimensional mitochondrial damage in AD.

Mitochondrial-targeted therapy provides a new pathway for repairing energy metabolism in AD. Multiple early clinical trials have shown safety and pathological improvement signals, but the certainty of cognitive benefits still requires validation in Phase III trials. Future efforts need to focus on delivery efficiency, combination therapy, and precise stratification to accelerate the translation from mechanism to clinical application.

A critical observation from the clinical studies discussed above is the striking disconnect between robust preclinical efficacy and consistently negative or inconclusive clinical outcomes. With the exception of biomarker signals in some trials, no mitochondrial-targeted agent has yet demonstrated definitive cognitive benefit in AD patients in Phase III settings. This translational gap likely reflects multiple factors: the use of animal models that inadequately recapitulate sporadic AD, the intervention window that falls far too late in the disease course, the lack of validated biomarkers to identify patients with prominent mitochondrial contributions to their pathology, and the fundamental limitation of monotherapy targeting a single mitochondrial node in a disease sustained by multiple interacting drivers. These observations should temper expectations and guide future trial designs toward earlier intervention, combination strategies, and biomarker-guided patient stratification.

## 8 Limitations, challenges and future prospects

Mitochondria-targeted therapy for AD represents a paradigm shift from traditional clearance of pathological proteins to repairing cellular energy metabolism, demonstrating preclinical promise. However, the translation process from breakthrough discoveries in basic research to clinical applications that ultimately benefit patients faces severe multidimensional and systematic challenges.

### 8.1 Core challenge: Barriers to translation from basic to clinical research

#### 8.1.1 Disjunction between rodent model and human clinical trial results

The field of AD drug development is facing an extremely high failure rate. Cummings team has systematically organized the global pipeline of investigational drugs for AD over consecutive years, quantitatively disclosing clinical translation data at each stage: According to statistics, between 2002 and 2012, there were a total of 413 AD clinical trials (124 Phase I, 206 Phase II, 83 Phase III), involving 244 independent candidate compounds. Only 0.4% of these ultimately achieved positive translatable efficacy, resulting in a failure rate of 99.6%., far exceeding that in other disease areas (Cummings et al., 2014). This indicator rose to 2.7% between 2012 and 2021 ((Cummings et al., 2022)). The latest 2025 pipeline report covering programs initiated between 2012 and 2024 shows that the translational success rate has further improved to approximately 4.0% (Cummings et al., 2025), directly reflecting the long-standing translational bottlenecks in the development of new AD drugs. That is, seemingly encouraging results in rodent models are often difficult to replicate in human clinical trials. Firstly, existing transgenic rodent models of AD are predominantly constructed based on rare familial AD mutations, making it difficult to simulate the multifactorial complex pathogenesis of sporadic Alzheimer’s disease, which accounts for over 95% of AD cases. Secondly, rodents lack the human-specific mitochondrial DNA haplogroup genetic heterogeneity, which can directly regulate mitochondrial function, oxidative stress, and inflammatory responses, leading to differences in drug response between humans and animals. Additionally, experimental animal intervention cycles are short, breeding environments are standardized, and genetic backgrounds are homogeneous, which prevent the simulation of long-term off-target organ toxicity that occurs in elderly Alzheimer’s disease patients after prolonged drug exposure.

#### 8.1.2 Mechanical complexity

The extent and mechanisms of mitochondrial damage vary significantly across different brain regions (hippocampus/cortex), cell types (neurons/glia), and disease stages, resulting in limited efficacy of single-target interventions. The mitochondrial functional network is extensive, involving multiple processes such as the respiratory chain, mtDNA repair, and autophagy. The interactions between targets are complex, and combination drug therapy regimens lack systematic validation.

#### 8.1.3 Technical bottlenecks- precise delivery and target specificity

The blood-brain barrier (BBB) strictly restricts the entry of most macromolecules and hydrophilic drugs into the central nervous system. Even if a drug successfully enters the brain, it must further penetrate the neuronal cell membrane and specifically accumulate in dysfunctional mitochondria, while avoiding impact on other normal organelles or cell types to prevent off-target toxicity. The current solution is highly dependent on innovative technologies such as intranasal delivery and intelligent nanodelivery systems (Qian et al., 2025; Na et al., 2026).

#### 8.1.4 Balance between safety and efficacy-long-term risks remain unclear

Mitochondria are the primary sites for the production of ROS. While short-term, moderate enhancement of mitochondrial activity may be beneficial, long-term or excessive stimulation can increase ROS production, exacerbate oxidative stress, and inflict secondary damage on already vulnerable neurons (Reddy, 2006). Mitochondria maintain a healthy network through continuous fission, fusion, and autophagy (mitochondrial quality control). Exogenous interventions may disrupt this delicate dynamic balance (Kakoty et al., 2025). AD is a highly heterogeneous disease. The patterns of mitochondrial damage may vary among different patients, across various disease stages, in distinct brain regions, and even among different cell types. A single ‘enhancement’ strategy may not be effective for all patients and could even be harmful to certain subgroups, suggesting a need for personalized approaches in the future. Furthermore, the inherent complexity of AD as a multifactorial disease, in which aging, genetics, vascular health, and systemic metabolism all contribute to disease onset and progression, means that no single pathogenic mechanism, including mitochondrial dysfunction, can fully explain the heterogeneity of clinical presentation and treatment response. This reality underscores the need for combination therapies and patient stratification strategies that acknowledge the variable contribution of different pathological drivers across individuals.

### 8.2 Future prospects: innovation strategies and integration pathways

#### 8.2.1 Development of intelligent, multi-level targeted delivery systems

Integrating technologies such as intranasal administration, lesion microenvironment-responsive nanoparticles, and cell/mitochondria-targeting peptides to achieve precise delivery that “crosses the BBB → accumulates in lesion brain regions → enters neurons → targets mitochondria.” (Qian et al., 2025).

#### 8.2.2 Exploring combination therapy and multi-target intervention

Given the complexity of AD and mitochondrial dysfunction, monotherapy may not suffice to contain the disease. Combining mitochondrial-targeted drugs with anti-inflammatory therapies, or traditional Aβ/tau-targeted therapies, may prove to be a more effective strategy. Researchers drew inspiration from animal hibernation. Hibernating animals are capable of rapidly and reversibly regulating mitochondrial function, switching between inhibition and activation without causing oxidative damage, which provides a novel biological blueprint for developing dual-targeted therapies that can both enhance ATP production and control ROS((de Veij Mestdagh et al., 2023)).

#### 8.2.3 Patient stratification and precision medicine using biomarkers

Identify specific biomarkers in blood or cerebrospinal fluid that reflect mitochondrial dysfunction (such as mtDNA copy number, oxidative damage products, or markers of specific pathway activity) to identify patient subgroups most likely to benefit from mitochondrial-targeted therapies, thereby enabling precision medicine. Integrating mtDNA haplogroup genetic typing for population stratification is a core future direction: cohort studies in middle-aged populations have confirmed that mtDNA haplogroups can long-term predict late-life cognitive decline trajectories, independent of traditional cognitive markers (Yu et al., 2025). For patients with high-risk haplogroups such as T, U, and K with AD, antioxidant and mitochondrial autophagy-promoting combined interventions should be initiated earlier. For protective haplogroup populations such as H, the focus can be on aging prevention and mild early intervention. Based on mitochondrial genetic background, medication regimens and treatment courses should be designed differentially to maximize the clinical benefits of MER therapy. It is important to note that haplogroup J exhibits risk heterogeneity among its subtypes (J1c subtype may increase risk, while J2 subtype is protective) (Liu H. et al., 2021). However, the overall epidemiological evidence from European populations classifies J as a protective haplogroup, consistent with reduced AD risk.

## 9 Discussion

This review, after integrating existing evidence, proposes a core view: mitochondria function as a critical hub within the AD pathological network, one that integrates metabolic, redox, and immune signals and engages in bidirectional crosstalk with Aβ and tau pathologies. Mitochondrial dysfunction is far more than just an imbalance in energy metabolism and excessive production of ROS. It converts intracellular metabolic crises into a systemic neuroinflammatory storm driving disease progression by releasing DAMPs, such as mtDNA. Specifically, mtDNA released by damaged mitochondria can be recognized by pattern recognition receptors (such as TLR9) within immune cells such as microglia, thereby activating the cGAS-STING pathway and the NLRP3 inflammasome. This leads to the massive release of pro-inflammatory cytokines, resulting in the formation of a persistent chronic inflammatory microenvironment. Abnormalities in this ‘metabolic-immune’ dialogue constitute a core link in the pathophysiology of AD. This may be the underlying theoretical basis for why mitochondrial-targeted therapy is superior to single-target approaches (such as Aβ clearance). MtDNA depletion in microglia of the brains of AD patients is significantly associated with disease staging and pathological susceptibility, suggesting that mitochondrial-mediated immune dysregulation is an early event in AD (13). More importantly, mitochondrial dysfunction forms a vicious cycle with the two core pathological proteins of AD—Aβ and tau protein. Therefore, mitochondria are no longer the endpoint downstream of the pathological cascade but serve as key amplifiers driving Aβ and tau pathologies, linking metabolic dysregulation with neuroinflammation (Wong et al., 2026). This hub position implies that mitochondrial-targeted therapies may be more fundamental than simply clearing Aβ or tau, as they aim to disrupt the central node where multiple pathological pathways converge, rather than merely addressing downstream pathological products.

Based on this, I propose a paradigm shift from single-target clearance to MER. This restoration process is not a one-dimensional intervention but a logically rigorous, step-by-step systematic project. As mentioned above, this strategy covers three progressive stages including clear, rebuild and consolidate. Phase I focuses on eliminating oxidative damage and restoring kinetic balance. Phase II focuses on activating endogenous biogenesis to rebuild functional reserves. Phase III focuses on blocking the mtDNA-triggered innate immune cascade to consolidate microenvironmental homeostasis. It should be noted that this stepwise repair strategy is not a simple assembly of drugs, but a rational design based on the temporal evolution characteristics of the pathological network.

It is critical to delineate MER from related concepts to establish its genuine conceptual advance (Table 3). The MER framework aims to address a unique question that existing theories cannot resolve: how to systematically restore the overall homeostasis of the mitochondrial ecosystem through phased, multi-targeted synergistic interventions, rather than merely correcting individual defects. Existing frameworks, including MQC, the mitochondrial cascade hypothesis, mitohormesis, and mitochondrial transplantation, each address limited aspects of mitochondrial dysfunction. However, none of them provides: A multidimensional, quantitative operational definition for what constitutes successful restoration of the mitochondrial ecosystem; A phased, temporally coordinated intervention logic that explicitly links each therapeutic step to specific measurable dimensions; and an integrated framework that simultaneously accounts for bioenergetic failure, oxidative stress, dynamic imbalance, mitophagy defects, and immune activation as interconnected rather than independent targets.

**TABLE 3:** Comparison of MER with existing mitochondrial frameworks.

Comparison dimension	Mitochondrial cascade hypothesis	MQC	Mitohormesis	Mitochondrial transplantation	MER framework
Objective	Identify mitochondria as the single upstream trigger of AD pathological lesions	Eliminate individual damaged mitochondrial organelles	Activate endogenous protective responses via mild adaptive stress stimuli	Replace defective mitochondria with exogenous healthy organelles	Systematically reconstruct the overall microecological function of neurons
Intervention Logic	Linear unidirectional pathological cascade chain	Local clearance of dysfunctional mitochondria	Passive stress preconditioning	Cell replacement therapy	Three sequential intervention phases: Cleaning Period → Reconstruction Period → Consolidation Period
Covered Dimensions	Single organelle injury only	Mitochondrial fission, fusion and mitophagy	Redox homeostasis only	Global restoration of mitochondrial pool function	Five dimensions: bioenergetics, redox balance, mitochondrial dynamics, quality control, metabolism-immune axis; covers upstream lesions including MAM dysfunction, calcium overload and mitochondrial proteostasis defects
Quantifiable Evaluation Markers	Single mitochondrial dysfunction biomarkers	Expression of quality-control proteins such as PINK1, Parkin and Drp1	ROS levels and antioxidant enzyme activity	Mitochondrial viability and ATP production	Five key biomarkers (Repair is defined when at least three indicators return to normal)
Key Limitations	Ignores bidirectional crosstalk between mitochondria, endoplasmic reticulum and immune system	Neglects inter-organelle communication and systemic neuroinflammation	Relies on low-dose stress induction and fails to reverse established severe pathological damage	Risks immune rejection, delivery barriers and invasive surgical operations	The “≥3/5”criterion is a provisional standard for preclinical screening and has not been calibrated in large clinical cohorts
Exclusive Innovations of MER	MER adopts a bidirectional network perspective integrating MAM structure, calcium homeostasis and proteostasis-neuroimmune axis, breaking linear pathological logic	MER expands repair scope to inter-organelle MAM networks and metabolism-immune axis, upgrading from single-organelle repair to holistic microecological restoration	MER employs active combinatorial drug therapy without stress priming to directly correct established pathological defects	MER activates autologous mitochondrial repair via BBB-permeable oral small molecules, featuring non-invasive administration and superior translational potential	Proposed staged multi-target intervention paradigm that simultaneously targets upstream MAM lesions and downstream neuroinflammation, together with quantifiable criteria for evaluating ecosystem-level repair

AD as a multifactorial network disease: It is important to situate the mitochondrial-centric perspective within the broader landscape of AD pathogenesis. Compelling evidence supports that AD arises from the convergence of multiple age-related insults including Aβ aggregation, tau hyperphosphorylation, chronic neuroinflammation, cerebrovascular dysfunction, and metabolic decline, none of which operates in isolation. These pathological processes are engaged in bidirectional crosstalk: Aβ can seed tau pathology and activate microglia; tau can exacerbate mitochondrial transport deficits; and mitochondrial dysfunction can drive both protein aggregation and innate immune activation through mtDNA release. The relative dominance of each mechanism likely shifts over the prodromal-to-dementia continuum and differs across individuals based on genetic background including APOE ε4 status and mtDNA haplogroups, sex, and comorbidities. This network view has profound therapeutic implications: targeting a single node, whether Aβ, tau, or mitochondria, may be insufficient to halt a disease sustained by multiple self-amplifying loops. Instead, effective disease-modifying strategies will likely require rationally designed combination regimens that address interconnected pathological drivers in a temporally coordinated manner. This principle aligns with the MER framework while acknowledging that mitochondrial restoration alone cannot be expected to fully reverse the neurodegenerative process. A critical appraisal of the evidence hierarchy in mitochondrial AD research: The field of mitochondrial-targeted therapies for AD faces a fundamental evidentiary challenge:a substantial gap between robust preclinical findings and clinical translation. A historical analysis of AD drug development reveals that between 2002 and 2012, the translational success rate was merely 0.4% (failure rate: 99.6%), which improved to approximately 4.0% for programs initiated between 2012 and 2024. While mitochondrial-targeted agents have not been systematically tracked in these metrics, the consistent failure of mechanistically diverse candidates (e.g., Dimebon, CoQ10, MitoQ in PD) suggests that this translational gap is not unique to Aβ-directed therapies.

Several factors contribute to this disconnect. First, the reproducibility of mitochondrial findings across laboratories is often compromised by differences in experimental conditions—mouse genetic background, diet, housing environment, circadian rhythms, and even gut microbiome composition—all of which significantly influence mitochondrial function and drug metabolism. Second, the methodological heterogeneity in assessing mitochondrial outcomes (bulk tissue vs. cell-type-specific assays; *ex vivo* measurements vs. *in vivo* imaging; acute vs. chronic intervention protocols) complicates cross-study comparisons and meta-analyses, making it difficult to aggregate evidence meaningfully. Third, the reliance on transgenic models based on rare familial AD mutations fails to capture the multifactorial etiology of sporadic AD, particularly the influence of human-specific mitochondrial DNA haplogroup diversity on drug response. Fourth, the interpretation of negative findings requires nuance. Failed clinical trials should not be simplistically interpreted as evidence against the mitochondrial hypothesis; rather, they provide critical lessons in patient stratification, intervention timing, and the limitations of monotherapy. For instance, the Dimebon failure suggests that transient mitochondrial membrane potential stabilization is insufficient without addressing upstream quality control deficits. Collectively, these observations underscore the urgent need for: (i) standardized reporting guidelines for mitochondrial endpoints in preclinical studies; (ii) adoption of human-relevant models (iPSC-derived neurons, cerebral organoids) that preserve human mitochondrial genetic backgrounds; (iii) biomarker-guided patient stratification to identify subgroups most likely to benefit; and (iv) rational combination therapies that target multiple nodes of the mitochondrial ecosystem rather than single pathways.

Although the MER framework provides a completely new logical system for AD treatment, it must be acknowledged its limitations. The “≥3/5 dimensions normalization” I propose is a heuristic starting point. The specific normalization threshold for each biomarker (e.g., how much of a decrease in 8-OHdG is considered to significant) requires rigorous experimental calibration in large-scale populations. Future research should use receiver operating characteristic (ROC) analysis to determine biomarker threshold that best correlate with cognitive endpoints. The current MER framework implicitly assumes applicability to all brain cells, which is its primary limitation. Existing evidence indicates that mitochondrial function varies significantly among neurons, microglia, and astrocytes. For example, activated microglial cells shift their metabolism toward glycolysis, whereas neurons strictly rely on oxidative phosphorylation (Cheng et al., 2021). The MER index of whole-brain homogenates may obscure opposing mitochondrial states in different cell types.

However, the clinical translation of the aforementioned staircase repair approach faces significant challenges. I propose that future breakthroughs will not lie in the discovery of more isolated drug molecules, but rather in the spatiotemporal coordinated delivery of multi-target drugs. Although mitochondria-targeted drugs (such as MitoQ, SS-31, etc.) have shown promise in improving cognition and reducing pathological markers in AD animal models, their performance in human clinical trials has repeatedly been disappointing. At the root of this implementation gap, there exists several critical underlying issues.

In the first place, the misalignment of the intervention window period is one of the core obstacles. Mitochondrial dysfunction is one of the earliest pathological events in AD, even preceding the appearance of Aβ plaques and neurofibrillary tangles. However, most current clinical trials enroll patients at an advanced stage of the disease, by which time there has been extensive neuronal loss and the mitochondrial network may have already sustained irreversible damage. Some studies have indicated that middle age may be the optimal window for intervention using mitochondrial-targeted compounds such as ginsenoside Re to extend lifespan and improve degenerative phenotypes (Jin et al., 2025). This requires that future clinical trial designs must be innovated. In the temporal dimension, therapeutic strategies must exhibit dynamic adaptability: during the early stages of AD (prior to or in the mild phase of Aβ deposition), treatment should focus on mitochondrial dynamics repair and biogenesis to preventively maintain energy homeostasis; whereas in the disease progression phase, when massive mtDNA release triggers a cytokine storm, treatment should instead prioritize immune modulation and clearance of damaged mitochondria (mitophagy).

In the second place, the last mile problem of drug delivery needs to be urgently addressed. Even if drugs can cross the BBB, their precise distribution among different cell types in the brain (neurons, astrocytes, microglia) remains a black box. As the primary drivers of neuroinflammation in AD, microglial mitochondrial metabolic reprogramming (shifting from oxidative phosphorylation to glycolysis) is a key feature of the pro-inflammatory phenotype (Li et al., 2022; Wong et al., 2026). However, it remains unclear whether existing drugs can specifically target and correct mitochondrial dysfunction in microglia. In the spatial dimension, it is imperative to address the challenges of uneven drug distribution within the brain and poor target specificity. In light of the nano-delivery systems mentioned above, such as T-CeNPs or micellar CTS, future strategies should use intelligent carriers to achieve efficient penetration of the BBB and precise targeting of neuronal mitochondria. This spatiotemporal coordinated strategy ensures that drugs with distinct mechanisms of action (such as antioxidants and anti-inflammatory agents) are released at the correct temporal and spatial sites to exert maximum synergistic effects.

Furthermore, to bridge the gap between basic research and clinical application, I propose that a precision mitochondrial medicine system guided by biomarkers must be established. Firstly, peripheral blood mtDNA-CN or oxidative damage products (such as 8-OHdG) should be utilized as real-time monitoring indicators to dynamically evaluate the recovery degree of mitochondrial function, thereby guiding individualized intervention windows. Secondly, considering the significant differences in mitochondrial metabolism among individuals with distinct genetic backgrounds (e.g., APOE ε4 carriers), future therapeutic strategies should be tailored based on patients’ genetic profiles.

Last but not least, the inherent limitations of animal models are a significant cause of translational failure. Common transgenic AD mouse models are overly reliant on mutations in a limited number of familial AD (FAD)-related genes, and thus cannot fully simulate the complex etiology of sporadic AD (SAD), which accounts for the majority of AD cases, especially the heterogeneity of human mitochondrial DNA haplogroup backgrounds. Laboratory environments which include noise, lighting, and caging conditions can significantly affect the physiological and epigenetic status of animals, interfering with the reliability and reproducibility of experimental results. More fundamentally, mice and humans differ significantly in genetics, physiology, and other aspects, which limits the ability of animal data to predict human responses. There should be a promotion of the adoption of new methodologies with greater human relevance (NAMs), such as neuronal models derived from human induced pluripotent stem cells (iPSCs), organoids, and organ chips, to complement or replace animal experiments.

For patients with advanced AD, the mitochondrial network within neurons may have undergone extensive failure, at which point drug interventions may be minimally effective. A more disruptive vision is mitochondrial transplantation or replacement therapy. Previous studies have attempted to transplant healthy exogenous mitochondria into damaged brain regions in AD mouse models and observed improvement in cognitive function (Li et al., 2022). In the future, combining bioengineering technologies, such as using healthy mitochondria derived from the patient’s own or allogeneic mesenchymal stem cells, or even developing ‘artificial mitochondria’ for transplantation, may become one of the potential therapeutic avenues to completely address the ‘energy depletion’ problem in neurons. Indeed, this poses substantial technical challenges, including transplant efficiency, immune rejection, and long-term safety issues.

## 10 Conclusion

Repositioning mitochondria as a central hub in the Alzheimer’s disease pathological network provides a compelling theoretical framework that deepens our mechanistic understanding and offers a rational basis for novel therapeutic strategies. However, the path from this conceptual advance to clinical benefit remains fraught with challenges. The MER framework proposed in this review should be viewed as a testable hypothesis-generating model, a roadmap for future investigation, rather than an established clinical paradigm. The MER theory proposed in this paper converts abstract mitochondrial protection mechanisms into quantifiable, phased intervention criteria and constructs a three-step combined therapeutic regimen of ‘clearance - reconstruction - consolidation’. Based on a systematic review of the mechanisms of mitochondrial damage in AD, targeted therapeutics, and translational challenges across the full text, this review distills three testable scientific hypotheses with operational feasibility, providing directional guidance for basic and clinical translational research. Hypothesis 1: Triple Therapy Synergy Hypothesis. Drp1 inhibitor (Mdivi-1) + SIRT1/PGC-1α activator (NR) + targeted antioxidant (MitoQ) triple combination will demonstrate synergistic efficacy in advanced preclinical models (e.g., 3xTg-AD or SAMP8 mice), achieving ≥3/5 MER criteria, and outperforming any monotherapy or dual therapy in cognitive improvement. Hypothesis 2: Haplogroup-guided intervention hypothesis. Individuals carrying high-risk haplotypes such as T, U, and K should undergo antioxidant combined with mitochondrial autophagy-promoting intervention at an earlier stage. For protective haplogroup populations such as H, the focus can be on aging prevention and mild early intervention. Hypothesis 3: Peripheral Biomarker Monitoring Hypothesis. MFI and circulating mitochondrial DNA copy number can serve as non-invasive dynamic biomarkers for real-time monitoring of mitochondrial repair levels following MER intervention.
